# Longitudinal profiles of plasma eicosanoids during pregnancy and size for gestational age at delivery: A nested case-control study

**DOI:** 10.1371/journal.pmed.1003271

**Published:** 2020-08-14

**Authors:** Barrett M. Welch, Alexander P. Keil, Thomas J. van ‘t Erve, Leesa J. Deterding, Jason G. Williams, Fred B. Lih, David E. Cantonwine, Thomas F. McElrath, Kelly K. Ferguson

**Affiliations:** 1 Epidemiology Branch, National Institute of Environmental Health Sciences, Research Triangle, North Carolina, United States of America; 2 Department of Epidemiology, University of North Carolina, Chapel Hill, North Carolina, United States of America; 3 Mass Spectrometry Research and Support Group, National Institute of Environmental Health Sciences, Research Triangle, North Carolina, United States of America; 4 Division of Maternal-Fetal Medicine, Brigham and Women’s Hospital, Harvard Medical School, Boston, Massachusetts, United States of America; National Institutes of Health, UNITED STATES

## Abstract

**Background:**

Inflammation during pregnancy is hypothesized to influence fetal growth. Eicosanoids, an important class of lipid mediators derived from polyunsaturated fatty acids, can act as both direct influences and biomarkers of inflammation through a variety of biological pathways. However, quantifying these distinct inflammatory pathways has proven difficult. We aimed to characterize a comprehensive panel of plasma eicosanoids longitudinally across gestation in pregnant women and to determine whether levels differed by infant size at delivery.

**Methods and findings:**

Our data come from a case–control study of 90 pregnant women nested within the LIFECODES prospective birth cohort study conducted at Brigham and Women’s Hospital in Boston, Massachusetts. This study included 31 women who delivered small for gestational age (SGA) babies (SGA, ≤10th percentile), 28 who delivered large for gestational age (LGA) babies (≥90th percentile), and 31 who delivered appropriate for gestational age (AGA) babies (controls, >10th to <90th percentile). All deliveries occurred between 2010 and 2017. Most participants were in their early 30s (median age: 33 years), of white (60%) or black (20%) race/ethnicity, and of normal pre-pregnancy BMI (median BMI: 23.5 kg/m^2^). Women provided non-fasting plasma samples during 3 prenatal study visits (at median 11, 25, and 35 weeks gestation) and were analyzed for a panel of eicosanoids. Eicosanoids were grouped by biosynthetic pathway, defined by (1) the fatty acid precursor, including linoleic acid (LA), arachidonic acid (AA), docosahexaenoic acid (DHA), or eicosapentaenoic acid (EPA), and (2) the enzyme group, including cyclooxygenase (COX), lipoxygenase (LOX), or cytochrome P450 (CYP). Additionally, the concentrations of the 4 fatty acids (LA, AA, DHA, and EPA) were measured in maternal plasma. Analytes represent lipids from non-esterified plasma. We examined correlations among eicosanoids and trajectories across pregnancy. Differences in longitudinal concentrations between case groups were examined using Bayesian linear mixed effects models, which included participant-specific random intercepts and penalized splines on gestational age. Results showed maternal plasma levels of eicosanoids and fatty acids generally followed U-shaped curve patterns across gestation. Bayesian models showed that associations between eicosanoids and case status varied by biosynthetic pathway. Eicosanoids derived from AA via the CYP and LOX biosynthetic pathways were positively associated with SGA. The adjusted mean concentration of 12-HETE, a LOX pathway product, was 56.2% higher (95% credible interval 6.6%, 119.1%) among SGA cases compared to AGA controls. Eicosanoid associations with LGA were mostly null, but negative associations were observed with eicosanoids derived from AA by LOX enzymes. The fatty acid precursors had estimated mean concentrations 41%–97% higher among SGA cases and 33%–39% lower among LGA cases compared to controls. Primary limitations of the study included the inability to explore the potential periods of susceptibility of eicosanoids on infant size due to limited sample size, along with the use of infant size at delivery instead of longitudinal ultrasound measures to estimate fetal growth.

**Conclusions:**

In this nested case–control study, we found that eicosanoids and fatty acids systematically change in maternal plasma over pregnancy. Eicosanoids from specific inflammation-related pathways were higher in mothers of SGA cases and mostly similar in mothers of LGA cases compared to controls. These findings can provide deeper insight into etiologic mechanisms of abnormal fetal growth outcomes.

## Abstract 2

In a cohort study, Barrett Welch and colleagues investigate associations between infant size for gestational age and plasma eicosanoids in pregnant women.

## Author summary

**Why was this study done?:**

- Inflammation during pregnancy is thought to influence fetal growth. Eicosanoids are an important class of lipid molecules derived from polyunsaturated fatty acids that can directly influence inflammation and be used as biomarkers of activity. This study characterized a large selection of these eicosanoids and fatty acids during pregnancy.
- Although some individual eicosanoids have been previously measured in pregnancy, this study provides a novel evaluation of a comprehensive eicosanoid panel for (1) changes over time in pregnant women and (2) associations with infant size for gestational age at delivery.

**What did the researchers do and find?:**

- Compared to mothers with infants of appropriate size for their gestational age (the control group), mothers of infants small for gestational age had higher blood plasma concentrations of eicosanoids related to inflammation. However, levels in mothers of large for gestational age infants were similar to those of the control group.

**What do these findings mean?:**

- This study provides additional evidence that inflammation processes related to lipid metabolism fluctuate over pregnancy. The differences in eicosanoid levels between mothers by infant size at delivery outcomes may provide further insights into mechanisms of disease and possible therapeutic targets.
- Future studies would benefit from a larger number of participants to evaluate differences in how eicosanoid levels change over time during pregnancy in cases compared to controls. Additionally, improving identification of cases and controls (e.g., with repeated ultrasound measurements) could improve assessment of these relationships.

## Introduction

Fetal growth disorders are major risk factors for adverse pregnancy and later life outcomes [1–4]. Proxy outcomes for disorders of fetal growth include small for gestational age (SGA, ≤10th percentile) and large for gestational age (LGA, ≥90th percentile) birth weight. Risk factors for SGA include maternal obstetrical complications, fetal genetic factors, infection, and various medical conditions [5]. LGA is a less studied outcome despite its increasing incidence in the United States and globally [6,7]. Major risk factors for LGA include maternal obesity, older age, hyperglycemia, and preexisting or gestational diabetes [4]. A large proportion of both SGA and LGA cases have unknown causes [5,8]. However, there is mounting evidence that drivers of inflammation and oxidative stress play an important role in fetal development [9–11].

Eicosanoids are a class of bioactive lipids produced from the oxygenation of polyunsaturated fatty acid precursors, including the primary omega-6 fatty acids (linoleic acid [LA] and arachidonic acid [AA]) and omega-3 fatty acids (docosahexaenoic acid [DHA] and eicosapentaenoic acid [EPA]) [12]. The biosynthesis of eicosanoids can occur enzymatically by cyclooxygenase (COX), cytochrome P450 (CYP), and lipoxygenase (LOX), or non-enzymatically via free radicals [12,13]. These lipid molecules are increasingly recognized as important vasoregulatory molecules and biomarkers involved in the pathophysiology of inflammation and oxidative stress [13,14]. There is growing evidence that eicosanoids are involved in a range of adverse pregnancy outcomes, including preeclampsia [15,16], preterm birth [17,18], and fetal growth disorders [19].

Recent advances in targeted lipidomics provide the ability to better characterize several eicosanoids from distinct biosynthetic pathways. However, few studies to date have investigated comprehensive panels of eicosanoids (i.e., including several biosynthetic pathways) during pregnancy [17,20,21], and none to our knowledge have examined patterns in eicosanoid levels across pregnancy. In this study we aimed to characterize repeated measurements of eicosanoids during pregnancy and assess associations with infant size at delivery (SGA and LGA) in a subset of women from an ongoing prospective birth cohort.

## Methods

### Study population

Women in this study were participants in LIFECODES, a prospective birth cohort with ongoing recruitment since 2006 that is conducted at Brigham and Women’s Hospital (BWH) in Boston, Massachusetts. Recruitment occurred in early gestation (<15 weeks), at which time women provided informed consent and completed demographic and medical history questionnaires. Non-fasting blood samples were provided at enrollment and up to 2 additional prenatal visits (at median 11, 25, and 35 weeks gestation). Women were eligible if they were carrying a non-anomalous fetus and planned to deliver at BWH. Gestational age estimation was performed according to the American College of Obstetricians and Gynecologists recommendations with the verification of last menstrual period by ultrasound [22]. Detailed information on pregnancy complications as well as gestational age and birth weight were recorded at delivery.

The present analysis utilizes a nested case–control study from within the LIFECODES population. Based on criteria established by Oken et al. [23], we defined SGA, LGA, and appropriate for gestational age (AGA) by birth weight percentiles for gestational age of ≤10th, ≥90th, and >10th to <90th, respectively. We randomly selected 30 SGA cases, 30 LGA cases, and 30 AGA controls that were frequency matched (1:1:1, SGA:AGA:LGA) based on the following criteria: maternal age (±5 years), maternal race/ethnicity (white, black, or other), maternal pre-pregnancy body mass index (BMI; ±5 kg/m^2^), and gestational age at delivery (±2 weeks). Women included in this study had singleton pregnancies between 2010 and 2017. Subsequent to participant selection and laboratory sample analysis, we identified an error in the coding of birth weight *z*-scores. This caused a total of 3 participants (3% of total sample) to have a misclassified case status that was subsequently changed for statistical analyses. Namely, 1 participant previously categorized as AGA was recategorized as SGA, and 2 participants previously categorized as LGA were recategorized as AGA. Thus, our final distribution of outcome groups was 31 SGA cases, 31 AGA controls, and 28 LGA cases. Of the 270 potential samples from 90 participants over 3 visits, a total of 25 (9%) samples were not quantified due to missing sample (13 samples) or quality control failure due to insufficient sample volume (12 samples) (S1 Appendix). The final sample size for this study was 89 participants, as 1 participant was dropped due to missing plasma samples at all visits.

Study participants provided written informed consent. The Institutional Review Board at BWH approved study protocols, and the National Institute of Environmental Health Sciences deemed the use of data and biological specimens for this study as “not human subjects research.” This study is reported as per the Strengthening the Reporting of Observational Studies in Epidemiology (STROBE) guidelines (S1 STROBE Checklist).

### Eicosanoid quantification

A panel of eicosanoids and fatty acids was measured in plasma samples by the Mass Spectrometry Research and Support Group at the National Institute of Environmental Health Sciences (Research Triangle, NC, US) in 2018. Non-esterified (free) eicosanoid and fatty acid levels were quantified by liquid chromatography with tandem mass spectrometry (LC-MS/MS). A detailed description of the analytical processes used to quantify lipid biomarkers is provided in S1 Appendix. For statistical analyses, we examined eicosanoids that were detected in ≥50% of all plasma samples analyzed. A total of 27 eicosanoids met this criterion while the other 30 were excluded (S1 Table and S2 Table, respectively).

**Fig 1:**
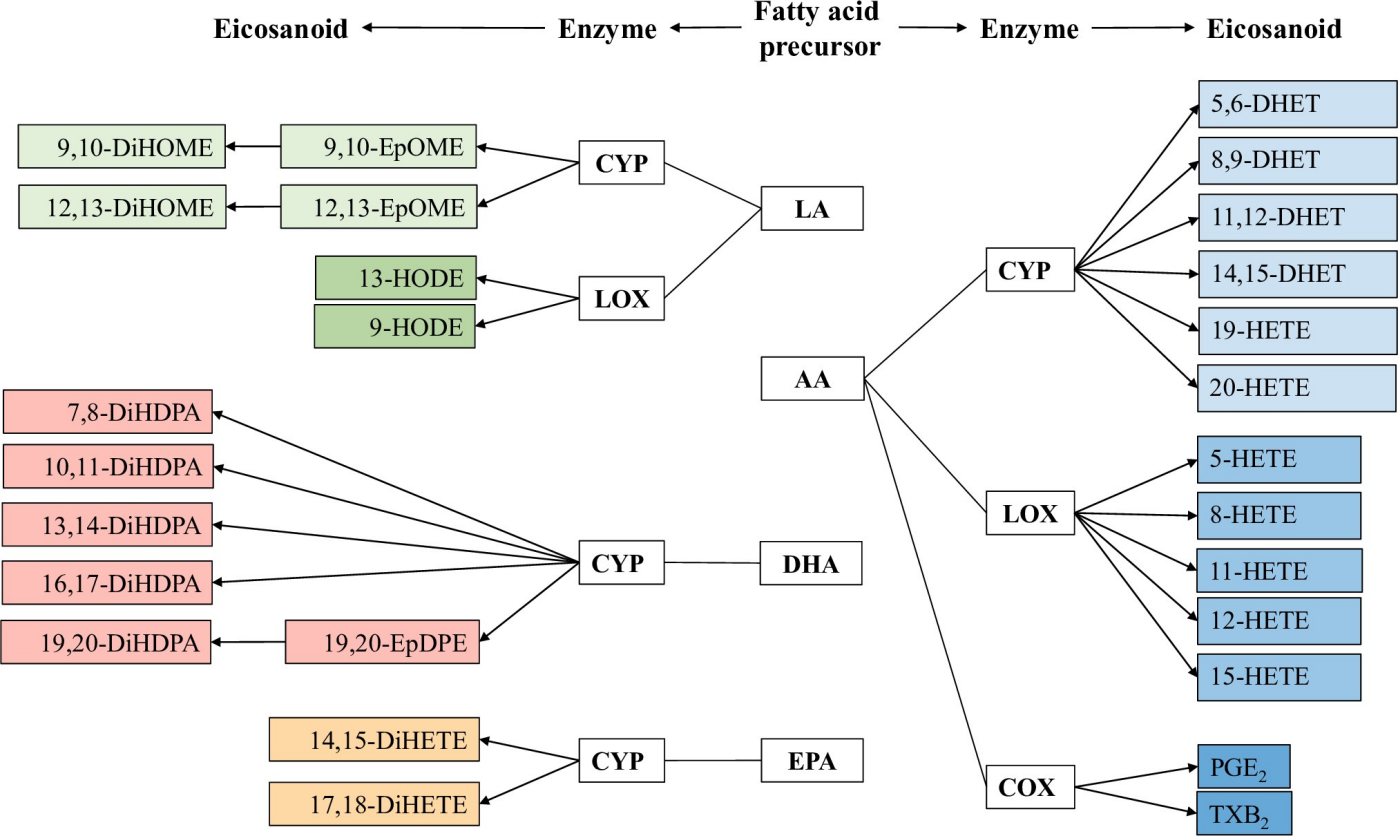
Primary biosynthetic pathways of eicosanoids. Schematic diagram for metabolic pathways of measured eicosanoids. The eicosanoid pathway is defined by a fatty acid precursor—linoleic acid (LA), arachidonic acid (AA), docosahexaenoic acid (DHA), or eicosapentaenoic acid (EPA)—that is metabolized by an enzyme—cytochrome P450 (CYP), lipoxygenase (LOX), or cyclooxygenase (COX). Colors represent the 7 possible eicosanoid groupings within this schema.

We grouped eicosanoids by primary known biosynthetic pathways of endogenous production (Fig 1), which considered the (1) precursor fatty acid and (2) enzyme group. The 4 fatty acid precursors were LA, AA, DHA, and EPA. The 3 enzyme groups were CYP, LOX, and COX. Based on these pathway criteria, we identified 7 fatty acid–enzyme groupings among eicosanoids meeting limit of detection (LOD) thresholds: LA-CYP, LA-LOX, AA-CYP, AA-LOX, AA-COX, DHA-CYP, and EPA-CYP. The full name and abbreviation of each eicosanoid is provided in S1 Table.

### Statistical analysis

Statistical analyses were performed using Stata version 15.1 (StataCorp, College Station, TX) and R version 3.5.2 [24] between 2019 and 2020. First, we examined study population characteristics for matched and non-matched variables. Differences in characteristics between case groups were examined using Kruskal–Wallis or chi-squared tests. We calculated median (interquartile range) eicosanoid concentrations overall and by prenatal study visit. To examine the variability in eicosanoid levels across gestation by participant, we calculated intraclass correlation coefficients (ICCs) and 95% confidence intervals in the overall population. ICC estimates were calculated for log_2_-transformed eicosanoid concentrations using maximum-likelihood random-effects models with Stata [25]. The ICC estimates provide an estimated ratio of between-individual to between- plus within-individual variability and can hold values that range from 0 to 1, with lower values indicating greater time-dependent variability [26]. Additionally, we visually assessed Spearman correlation coefficients between log_2_-transformed concentrations at each prenatal study visit using heatmaps.

We further examined changes in eicosanoid levels over gestation and assessed associations with infant size at delivery categories using Bayesian linear mixed effects models (BLMs) within the R package *brms* (version 2.10.0) [27]. This longitudinal modeling approach can easily handle missing values of predictors between timepoints. Briefly, the model for each eicosanoid at gestational age *t* for individual *i* is given as:

$$\log_{2}\left({\bar{x}}_{it}\right)=\beta_{0}+\beta_{1}LGA+\beta_{2}SGA+\beta_{3}Z+s(t)+b_{i}+\epsilon_{it}$$

where ϵ_*it*_ ~ normal (0,σ^2^); $\log_{2}\left({\bar{x}}_{it}\right)$ is the log (base 2)-transformed, centered eicosanoid concentration; *Z* is a covariate set; *b*_*i*_ is a participant-specific random intercept to account for repeated sampling; and *s*(*t*) is a smoothing spline with 10 knots on gestational age at sample collection to allow flexible assessment of longitudinal profiles. The hierarchical Bayesian specification of this model is completed by placing a normal (0,1) prior on the model fixed effects β_0_, β_1_, β_2_, and β_3_; giving *b_i_* a normal prior with a common variance across individuals; and penalizing the spline coefficients by modeling them as random effects. Each BLM included fixed effects for the indicator variables of SGA and LGA as explanatory variables. Unadjusted BLMs included *s*(*t*), but did not include the covariate *Z*. Adjusted BLMs included the following covariates selected a priori: maternal age at enrollment (examined continuously), maternal race/ethnicity (non-Hispanic white [white], black, other), pre-pregnancy BMI (examined continuously), and insurance status at enrollment (private insurance plan or publicly provided health plan). The “other” race/ethnicity category was constructed as a condensation of larger categories with insufficient sample size to disaggregate for this study, including those reporting as Hispanic/Latino ethnicity or one of the following categories: South Asian, East Asian, Native American, Pacific Islander, multiracial, other, or unsure. Covariates were selected if they were used for case–control matching or if they were hypothesized to be confounders.

Following model fitting, predicted values of each eicosanoid for all individuals were calculated across the range of gestational age, yielding a marginal association of gestational age with eicosanoid levels, standardized to the distribution of covariates in the study sample. Marginal predictions were used for plotting longitudinal profiles and represent controls at referent or mean levels of all covariates: 33 years of age, white race, pre-pregnancy BMI of 25.2 kg/m^2^, and private health insurance. These marginal associations of gestational age were plotted to visualize time-dependent changes in eicosanoids over gestation, an approach that assumes parallel slopes between case groups. The parallel slopes assumption was evaluated by rerunning eicosanoid models stratified by case status and plotting the marginal association within each case group. To assess differences in eicosanoid concentrations by case status, we also estimated standardized population mean eicosanoid levels across gestation for each case group. To quantify differences in eicosanoid levels between case groups, we calculated the percent difference in standardized population mean levels and 95% credible intervals (CrIs) in cases (SGA or LGA) compared to AGA controls (referent category). In contrast to frequentist approaches (e.g., standard maximum likelihood), Bayesian analyses draw inference from posterior distributions and do not produce 95% confidence intervals or *p-*values. We provide additional details regarding our Bayesian approach, including prior distribution specifications, number of iterations, model control parameters, model fit criteria, and R code, in S2 Appendix.

Our primary sensitivity analyses were to examine the influence of maternal diet and concurrent comorbidity on associations between eicosanoids and infant size at delivery. To determine the potential influence of maternal diet, we examined the association of case group with a ratio of measured fatty acid precursors (i.e., ∑[LA + AA]/∑[DHA + EPA]). This ratio of composite levels, or sums, is an objective way to determine the relative balance of omega-6 (i.e., LA and AA) versus omega-3 (i.e., DHA and EPA) essential fatty acids in the maternal diet [28]. To determine the potential influence of maternal comorbidity, we excluded any women with a concurrent diagnosis potentially relevant to maternal inflammation or delivery size (*n =* 13). Concurrent diagnoses that prompted exclusion were chronic hypertension (*n =* 3), gestational hypertension (*n =* 4), preeclampsia (*n =* 1), gestational diabetes mellitus (*n =* 1), or preterm birth (*n =* 4). Additionally, we excluded women who reported smoking at enrollment (*n =* 6) as smoking can potentially influence lipid peroxidation [29]. Women with a concurrent comorbidity diagnosis or smoking history were only excluded in sensitivity analyses and not within primary analyses.

## Results

### Participant characteristics

Matched characteristics were similar between case groups (Table 1). No significant differences in matching characteristics between case groups were observed (*p* > 0.35), with the exception of gestational age at delivery (*p* < 0.05). Most women were in their early 30s, were white, and had normal pre-pregnancy BMI (median: 23.5 kg/m^2^). Gestational age at delivery was slightly lower among SGA cases compared to AGA controls or LGA cases. However, the mean difference was only 0.9 weeks for SGA cases compared to controls and 0.3 weeks for LGA cases compared to controls, which were both well within the 2-week window used for matching. The earliest gestational age at delivery was 36.6 weeks, and 4 women experienced preterm birth (i.e., delivered at <37 weeks gestation). Non-matched characteristics were also similar between case groups (Table 1). Most women had attended or graduated from college, had private health insurance, and were multiparous. There were no systematic differences in the distributions of variables used for matching between participants missing and not missing plasma samples (S7 Table).

**Table 1:** Study population characteristics by case–control status.

Characteristic	Overall study population (*N =* 90)	Small for gestational age cases (*n =* 31)	Appropriate for gestational age controls (*n =* 31)	Large for gestational age cases (*n =* 28)
**Matched variables**	*** ***	*** ***	*** ***	*** ***
Maternal age (years)	33.2 (29.5, 37.3)	33.6 (26.9, 37.3)	32.4 (30.4, 37.5)	34.4 (30.4, 37.1)
Maternal race/ethnicity				
White	54 (60)	19 (61)	18 (58)	17 (61)
Black	18 (20)	6 (19)	6 (19)	6 (21)
Other*	18 (20)	6 (19)	7 (23)	5 (18)
Pre-pregnancy BMI (kg/m^2^)	23.5 (21.4, 27.5)	23.0 (20.2, 27.9)	23.0 (22.3, 27.4)	25.7 (22.1, 27.9)
Gestational age at delivery (weeks)	38.7 (37.9, 39.4)	37.9 (37.1, 38.6)	39.0 (38.3, 39.6)	39.1 (38.8, 39.8)
**Non-matched variables**	*** ***	*** ***	*** ***	*** ***
Gestational age at sample collection (weeks)				
Visit 1	11.1 (9.0, 12.9)	10.3 (8.9, 11.7)	11.6 (8.9, 12.7)	11.1 (9.1, 16.4)
Visit 2	25.6 (24.9, 26.3)	25.4 (24.9, 26.0)	25.9 (24.9, 26.9)	25.4 (24.8, 26.3)
Visit 3	35.0 (34.4, 35.7)	34.6 (34.4, 35.6)	35.3 (34.4, 35.9)	35.3 (34.4, 35.9)
Birth weight (kg)	3.2 (2.4, 4.0)	2.3 (2.2, 2.4)	3.2 (3.0, 3.4)	4.2 (4.1, 4.3)
Birth weight percentile (%)	35 (4, 92)	3 (1, 4)	35 (23, 46)	96 (93, 98)
Health insurance				
Private	66 (73)	22 (71)	24 (77)	20 (71)
Public	24 (27)	9 (29)	7 (23)	8 (29)
Maternal education				
High school or less	7 (8)	3 (10)	3 (10)	1 (4)
Some college/technical school	22 (24)	8 (26)	8 (26)	6 (21)
College graduate or more	61 (68)	20 (65)	20 (65)	21 (75)
Parity				
Nulliparous	24 (27)	7 (23)	10 (32)	7 (25)
Parous	66 (73)	24 (77)	21 (68)	21 (75)
Smoking during pregnancy (yes)	6 (7)	4 (13)	2 (6)	0 (0)

Data are given as *n* (%) or median (25th, 75th percentiles).

*The “other” category is a condensation of larger categories with insufficient sample size for disaggregation (e.g., Hispanic/Latino ethnicity, South Asian, East Asian, Native American, multiracial).

### Eicosanoid variation over pregnancy

**Fig 2:**
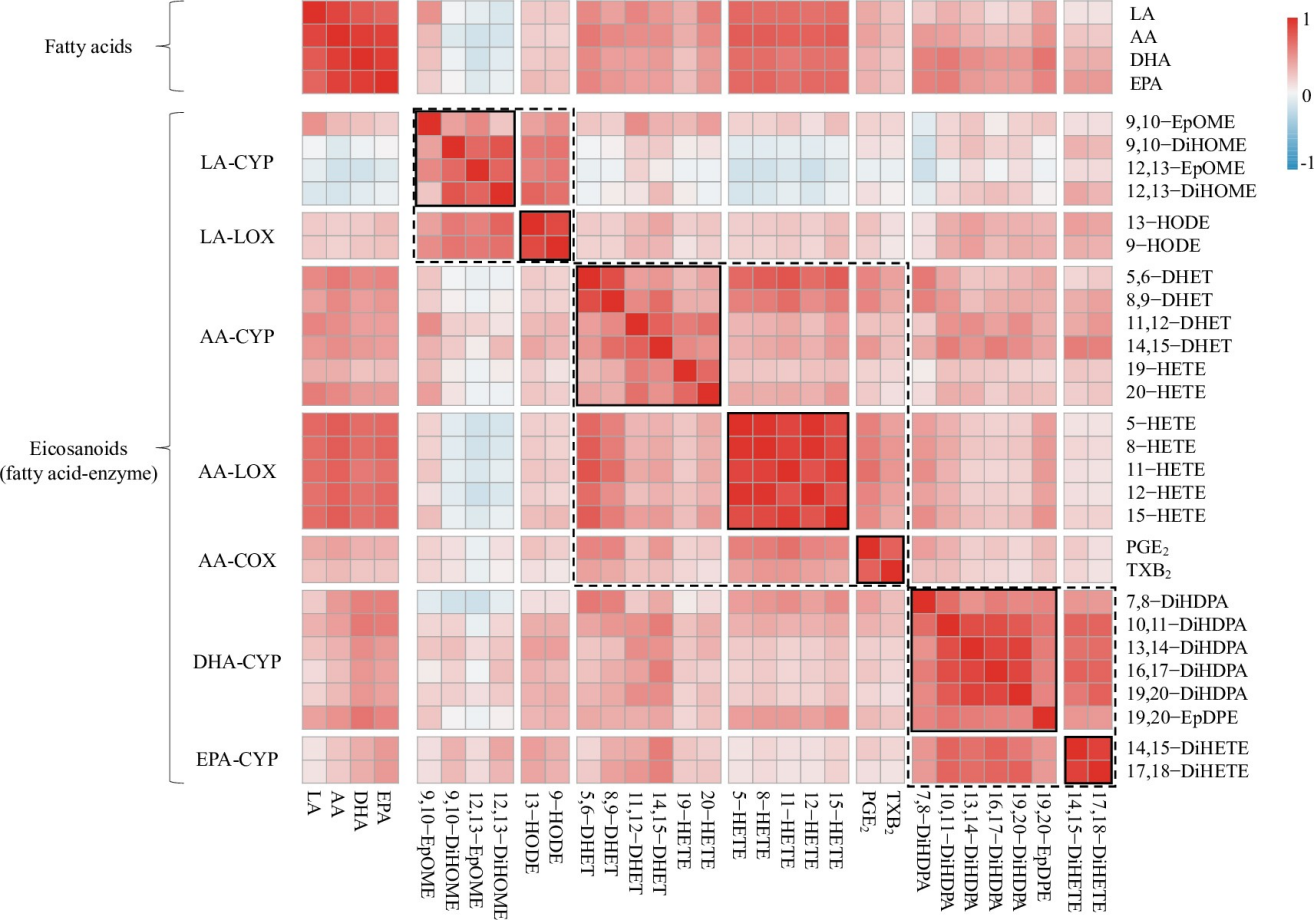
Spearman correlations between fatty acid and eicosanoid concentrations in early pregnancy maternal plasma (*n =* 89). Eicosanoid metabolites are grouped by biosynthetic pathway, which includes the fatty acid precursor and enzyme. Dotted black lines signify eicosanoid groups sharing a common fatty acid precursor, while solid black lines signify eicosanoid groups that also share a common enzyme pathway. AA, arachidonic acid; COX, cyclooxygenase; CYP, cytochrome P450; DHA, docosahexaenoic acid; EPA, eicosapentaenoic acid; LA, linoleic acid; LOX, lipoxygenase.

Eicosanoids were hypothesized to share a biosynthetic pathway if produced from the same fatty acid precursor and enzyme pathway, which included 7 fatty acid–enzyme groupings (Fig 1). Correlation patterns of eicosanoids measured at visit 1 supported this hypothesis (Fig 2). The strongest correlations were observed between eicosanoids sharing both a fatty acid precursor and enzyme, but modest correlations were also observed between eicosanoids only sharing a fatty acid precursor. Eicosanoids derived from the AA-LOX pathway (i.e., 5-, 8-, 11-, 12-, and 15-HETE) displayed the strongest correlations (Spearman correlation coefficient range: 0.81 to 0.97). This group of eicosanoids also had the strongest relative correlation with the plasma concentrations of fatty acid precursors (Spearman correlation coefficient range: 0.63 to 0.77). Conversely, other groups of eicosanoids had low to moderate correlations with fatty acid precursor concentrations. Correlation patterns were consistent for plasma samples taken at visits 2 and 3 (S1 Fig).

Plasma samples at prenatal visits 1, 2, and 3 were available for 80, 86, and 79 women, respectively (S3 Table). The fatty acid precursor with the highest abundance was LA, which corresponded to higher LA-derived eicosanoid concentrations relative to other eicosanoids. EPA had the lowest measured concentrations among fatty acid precursors, and most EPA-derived eicosanoids had low detection rates (S2 Table). The median concentrations of most markers tended to follow a U-shaped curve across gestational age, as the lowest values were measured at visit 2 during mid-pregnancy. Variability in concentrations by study visit was also reflected in estimated ICC values (S3 Table). The greatest variability in concentrations between study visits was observed for eicosanoids derived from LA-CYP (ICC range: 0.03–0.18). The variability between study visits for the remaining eicosanoids, which had lower median levels, was more modest (ICC range: 0.16–0.50).

**Fig 3:**
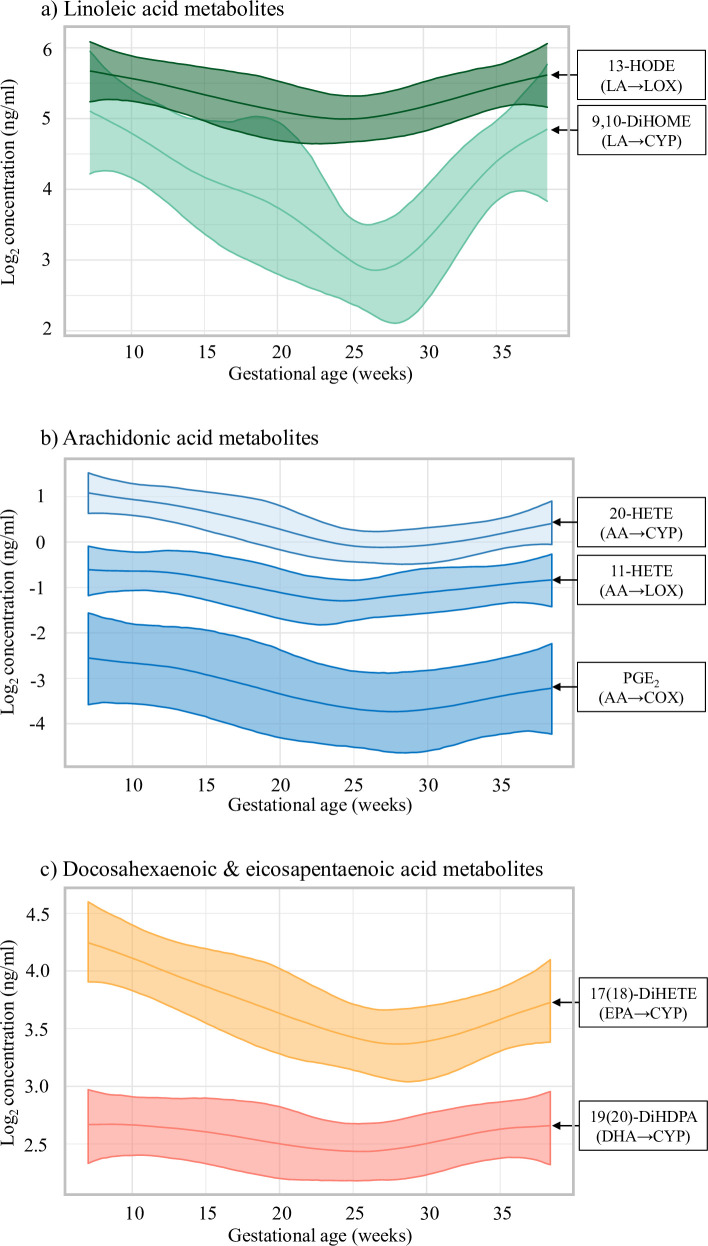
Model-predicted longitudinal profiles in eicosanoids over gestation in maternal plasma (*n =* 89). Longitudinal profiles represent standardized population mean concentrations and 95% credible intervals estimated by Bayesian linear mixed effects models, adjusting for maternal age, race/ethnicity, pre-pregnancy BMI, and health insurance status. Eicosanoid profiles are grouped by fatty acid precursors, including (a) linoleic acid (LA), (b) arachidonic acid (AA), and (c) docosahexaenoic acid (DHA) or eicosapentaenoic acid (EPA). Individual profiles are labeled with the name of the eicosanoid and the fatty acid–enzyme biosynthetic pathway, including the enzymes cytochrome P450 (CYP), lipoxygenase (LOX), or cyclooxygenase (COX).

Longitudinal changes in eicosanoid levels were also observed when modeled as a function of gestational age at sampling using BLMs (Fig 3). The predicted levels of eicosanoids derived from LA-CYP, such as 9,10-DiHOME, were most variable between early and late gestation (Fig 3A), while predicted levels for eicosanoids derived from AA, DHA, or EPA were generally more stable (Fig 3B and 3C). The assumption of parallel slopes between case groups was validated by models stratified by case group, as profiles were generally similar between controls and cases (S3 Fig). Evaluation of longitudinal profiles of fatty acid precursors showed similar patterns as observed with eicosanoids (S2 Fig). EPA plasma level seemed to have the most pronounced decrease between visit 1 and visit 2, and remained low in visit 3, while the other fatty acids had similar levels between visits 1 and 3.

### Eicosanoid differences by case status

**Fig 4:**
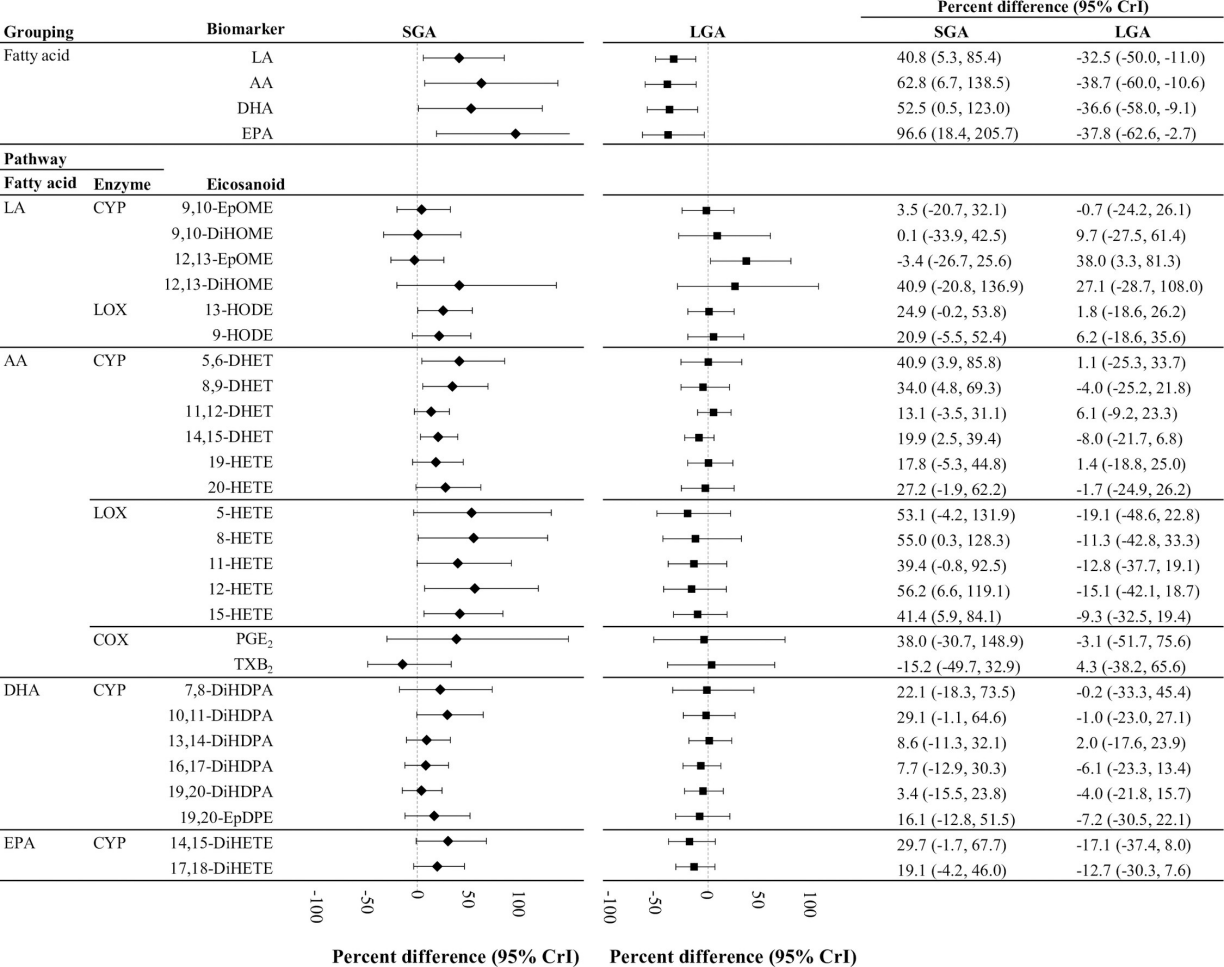
Percent differences in eicosanoid concentrations between women who delivered SGA infants or LGA infants compared to controls (*n =* 89). Percent differences and 95% credible intervals of longitudinal concentrations were generated by Bayesian linear mixed effects models that were adjusted for maternal age (continuous), race/ethnicity (white, black, other), pre-pregnancy BMI (continuous), and insurance status (private, public). Model estimates were transformed to percent differences in standardized population mean concentrations between SGA or LGA cases and appropriate for gestational age controls. AA, arachidonic acid; COX, cyclooxygenase; CrI, credible interval; CYP, cytochrome P450; DHA, docosahexaenoic acid; EPA, eicosapentaenoic acid; LA, linoleic acid; LGA, large for gestational age; LOX, lipoxygenase; SGA, small for gestational age.

Overall, compared to controls, the maternal plasma levels of fatty acids and eicosanoids tended to be higher in SGA cases and lower in LGA cases when evaluated using BLMs (Fig 4). Strong associations were observed between fatty acid precursors and case group. The standardized population mean concentrations of fatty acid precursors were 41%–97% higher among SGA cases and 33%–39% lower among LGA cases compared to controls. Although median EPA concentrations were the lowest among the fatty acid precursors, they displayed the strongest association with case status. The adjusted population mean level of EPA was 96.6% higher (95% CrI 18.4%, 205.7%) among SGA cases and 37.8% lower (95% CrI −62.6%, −2.7%) among LGA cases compared to controls.

Eicosanoid associations with case status varied between the 7 hypothesized biosynthetic pathways (Fig 4). The strongest associations with SGA case status were observed among eicosanoids produced from AA-CYP or AA-LOX pathways. The eicosanoid with the strongest association was 12-HETE, a product of the AA-LOX pathway, which had an adjusted population mean level that was 56.2% higher (95% CrI 6.6%, 119.1%) among SGA cases compared to AGA controls. We observed positive, but not statistically significant associations of SGA status with 2 other eicosanoid groups, including the HODEs produced from LA-LOX (range of mean difference: 21%–25% higher) and the DiHETEs produced from EPA-CYP (range of mean difference: 19%–30% higher).

Eicosanoid levels were generally lower among LGA cases versus controls, but the absolute difference was generally smaller than the difference between SGA cases and controls (Fig 4). Similar to SGA cases, the strongest associations among LGA cases were observed for the group of eicosanoids produced from AA-LOX (range of mean difference: 9%–19% lower). The eicosanoid with the strongest, but not statistically significant association among this group was 5-HETE, which had an adjusted population mean level 19.1% lower (95% CrI −48.6%, 22.8%) among LGA cases compared to AGA controls. Additionally, LGA cases had a suggestive negative association with the DiHETE eicosanoids produced from EPA-CYP (range of mean difference: 13%–17%). We observed null associations of case status with eicosanoids produced from AA-COX (PGE_2_ and TXB_2_) and DHA-CYP (DiHDPAs and 19,20-EpDPE).

Our sensitivity analyses revealed that results were consistent in models that were not adjusted for confounders (S4 Table), were restricted to women without concurrent comorbidities (S5 Table), or excluded women who reported smoking during enrollment (S6 Table). Additionally, sensitivity analyses provided evidence that results were not influenced by systematic differences in maternal diet, which was assessed using the ratio measure of plasma fatty acids (i.e., ∑[LA + AA]/∑[DHA + EPA]). The ratio of composite levels had null associations with infant size at delivery categories, even though this ratio was 10.9% lower (95% CrI −29.2%, 11%) in SGA cases and 6.2% higher (95% CrI −15.1%, 31.3%) in LGA cases compared to controls in adjusted BLMs.

## Discussion

To our knowledge, this study provides a novel longitudinal characterization of a comprehensive panel of eicosanoids across pregnancy. We grouped eicosanoids based on extensive evidence of the major fatty acid and enzymatic pathways [12,30]; this grouping approach is supported by the correlation structure and longitudinal profiles we observed in our study population. Our results suggest that maternal concentrations of eicosanoids tend to follow a U-shaped curve between early and late pregnancy, with early gestation representing the period with the highest relative concentrations. Results also suggest that fatty acids in maternal plasma follow a similar gestational pattern as eicosanoids, but with less overall variability. The similarity in longitudinal profiles between eicosanoids and fatty acids is expected, as fatty acid metabolism directly influences eicosanoid production [12]. Our observations are consistent with epidemiologic evidence of changes in fatty acid levels across pregnancy [31–34]. However, this comparison must be interpreted cautiously, as these prior studies primarily quantified esterified or total fatty acids and we measured free fatty acid levels. Over the course of pregnancy, there are natural fluctuations in maternal metabolism of fatty acids that help promote healthy placental function and fetal growth [35]. The decrease in circulating maternal fatty acids we observed during mid-pregnancy (around 20 weeks gestation) corresponds to the estimated period when fetal uptake of these fatty acids dramatically increases [36]. The relatively higher levels of eicosanoids we observed in early pregnancy indicate this window may represent a relatively more susceptible period for the influence of eicosanoids on maternal inflammation and fetal growth.

### Eicosanoid associations with infant size at delivery

Importantly, we find that, compared to AGA controls, mothers who deliver SGA babies have higher eicosanoid levels, while mothers who deliver LGA babies have lower levels of a smaller set of eicosanoids. The strongest associations with SGA are among AA-derived eicosanoids, which is most evident for those produced by LOX enzymes (i.e., HETEs). Increased activity of LOX pathways has been implicated in inflammation-mediated outcomes during pregnancy, such as hypertension, preeclampsia, and spontaneous preterm birth [17,19,37]. However, we believe our results are independent of such complications as associations were consistent after excluding women with concurrent comorbidities (*n =* 13) (S5 Table). Our results indicate that women who deliver SGA infants may have higher LOX-related inflammation compared to controls, whereas this inflammatory pathway is not detected among women who deliver LGA infants.

The other group of AA-derived eicosanoids we found to have relatively strong associations with SGA were those produced by CYP (i.e., DHETs and HETEs). Elevated levels of DHETs in maternal blood have also been associated with pregnancy complications like preeclampsia and preterm birth [15,17]. DHETs are downstream products of epoxyeicosatrienoic acids (EETs), which are rapidly metabolized and thus detected at only low levels [12]. Thus, DHETs may also reflect EET levels in pregnancy. Since EETs are considered to be anti-inflammatory [13], the higher levels of DHETs we observe may indicate elevated anti-inflammatory response to systemic pro-inflammatory CYP activity.

The positive associations we observed of CYP-related HETEs with SGA status are important as these eicosanoids play important roles in vasoregulation [12]. The role of these eicosanoids during human pregnancy is unclear, as only a few epidemiologic studies have examined 20-HETE, and they found null cross-sectional associations with pregnancy complications like preeclampsia and preterm birth [15,17]. However, 20-HETE is a potent promoter of hypertension and has been associated with a range of adverse cardiovascular outcomes and been a target for therapeutic antihypertension drugs [38,39]. Our study provides novel evidence that these CYP-related HETEs may also influence the inflammatory process in mothers who deliver SGA infants.

We did not observe associations between infant size at delivery and the AA-derived eicosanoids of COX enzymes, which include PGE_2_ and TXB_2_. Prostaglandins like PGE_2_, and to a lesser extent thromboxanes like TXB_2_, have been recognized as important mediators of pregnancy outcomes and as being of particular importance around the time of delivery [40–43]. Recent studies have found positive associations between cord blood concentrations of PGE_2_ and TXB_2_ and the length of gestation for both term and preterm pregnancies [18,20]. Therefore, it is possible that these eicosanoids have greater relevance for the timing of parturition than for fetal growth. Alternatively, because COX metabolism is rapid and localized, urine may be a better matrix than plasma for measuring these eicosanoids [13,44]. However, there is still controversy as to whether COX-derived eicosanoids measured in urine can represent production related to underlying inflammation in the whole body versus specific to kidney [45,46].

Our results provide evidence of an association between infant size at delivery and plasma concentrations of fatty acid precursors, which represent the major circulating omega-6 and omega-3 polyunsaturated fatty acids. The higher levels of free fatty acids we observed among SGA cases compared to AGA controls is consistent with several previous studies [47,48], though those studies measured concentrations during postpartum periods or within different sample matrices (whole blood). However, other studies have found opposing or null associations with study designs that varied by timing (i.e., at delivery or postpartum) and matrix (e.g., erythrocytes or whole blood) of sampling [33,34,49]. The lower levels of fatty acids we observed among LGA cases compared to AGA controls is also consistent with prior studies [34,50,51]. A potential interpretation of the differences within maternal plasma by case status is that, compared to AGA fetuses, SGA and LGA fetuses are utilizing fatty acids at lower and higher rates, respectively [36,48]. The interpretation of our results regarding fatty acids must be made carefully, as we measured free, or non-esterified, levels, which represent a relatively small portion of total fatty acids in plasma because most are present in esterified form [52].

### Strengths and limitations

The primary strength of our study was the repeated sampling of mothers over key periods of pregnancy, which allowed us to perform novel longitudinal characterizations of a large panel of fatty acid precursors and eicosanoids. Additionally, we investigated a comprehensive panel of these compounds, as has only been examined in 1 other population of pregnant women [17], which allowed us to see differences in multiple eicosanoid pathways simultaneously.

We recognize that our study also had a number of limitations. First, we were limited by a relatively small sample size, which restricted our ability to explore possible joint associations between eicosanoids, or periods of susceptibility for eicosanoid influence on infant size at delivery. However, models stratified by case group support our assumption of parallel slopes within the primary statistical models. Additionally, our matched case–control study design allowed more efficient estimates—given the necessarily small sample size—that were not due to maternal characteristics such as age, race/ethnicity, and pre-pregnancy BMI. While matching allows precise characterization of certain estimates, it limits our ability to evaluate questions about prospective relationships such as addressing periods of susceptibility to eicosanoids. Due to this restriction, we were unable to estimate differences in eicosanoids and growth outcomes by study visit with acceptable precision. These potential periods of susceptibility will be interesting to explore in a larger sample. Second, the localized nature of eicosanoid biosynthesis and bioactivity reduces our ability to infer differences between specific tissues (e.g., placenta versus vascular endothelium) [13].

Third, the availability of a large number of eicosanoids and paucity of prior epidemiologic research necessitated our approach to analysis, which involved fitting a large number of statistical models. In such settings, hypothesis testing is unreliable due to issues of multiple comparisons and generally results in overestimates of statistical significance. We obviate this issue somewhat through the use of Bayesian modeling approaches, which tend to reduce issues of multiple comparisons provided that one can specify reasonable and informative prior distributions [53,54]. Further, we leveraged a priori knowledge to group eicosanoids by precursor fatty acids and enzyme group to assess whether findings were consistent within each biosynthetic pathway. This approach does not emphasize the use of null hypothesis testing for ranking the importance of results.

Additionally, it is important to note that we measured free, or non-esterified, levels of eicosanoids and fatty acid precursors in plasma samples. We feel our use of non-esterified lipid portions is justified given that the primary aim of this study is to assess associations with eicosanoid inflammation pathways, as non-esterified eicosanoids are typically considered more biologically relevant for inflammatory processes [13]. However, this approach may limit comparisons with other studies that measure esterified eicosanoids or polyunsaturated fatty acids. Although plasma concentrations of free fatty acids can accurately approximate levels in adipose tissue when a fasting blood sample is taken [52], our participants provided non-fasting plasma samples. Thus, we cannot determine the degree to which quantified concentrations of free polyunsaturated fatty acids represent transient levels due to a recent meal. Although we did not have data on maternal diet or supplementation habits during pregnancy, we believe that systematic differences in recent dietary intake between cases and controls is unlikely. We observed null associations between infant size at delivery categories and our proxy measure of polyunsaturated fatty acid intake (i.e., plasma ratio of omega-6 to omega-3 fatty acids), which provides an objective measure of maternal fatty acid intake [28]. This indicates that mothers of SGA, LGA, and AGA babies were unlikely to have systematic differences in fatty acid intake, which is consistent with prior epidemiologic studies that have evaluated associations between this ratio and infant size at delivery [33,48]. When feasible, future studies should quantify eicosanoids and precursor polyunsaturated fatty acids in both esterified and free forms to improve determination of their relative contributions to health outcomes.

Along with diet, we were limited by a lack of information on medication use (e.g., low-dose aspirin), which could potentially influence maternal eicosanoid levels. Low-dose aspirin would likely alter certain eicosanoid pathways (e.g., COX), but its use is not recommended for pregnant women unless they are at high risk of developing preeclampsia [55]. We had relatively few participants with comorbidities potentially relevant to receiving such a prescription, and excluding them from analyses did not alter results, thus we feel medication use is unlikely to influence our results.

Finally, we utilized SGA and LGA to approximate fetal growth in our study, which are less clinically relevant than fetal growth restriction and macrosomia. This was done for simplicity for the present study, but future work should utilize additional prenatal measures (e.g., ultrasound measurements) to define fetal growth outcomes. Combining a larger sample size with ultrasound measurements could also allow future studies to assess the value of eicosanoids for predicting restricted or excess fetal growth.

## Conclusions

In a nested case–control study population, we observed longitudinal patterns in fatty acid precursors and eicosanoids over pregnancy. Fetal growth outcomes, including SGA and LGA, were strongly associated with fatty acids precursors, with higher levels among mothers who delivered SGA babies and lower levels among those who delivered LGA babies. We also observed unique associations between plasma eicosanoids and fetal growth outcomes that were consistent across biosynthetic pathways. Eicosanoids representing potential inflammatory processes were higher in SGA cases compared to AGA controls, which was most evident for eicosanoids derived from pathways involving AA and LOX or CYP enzymes. Conversely, eicosanoid levels were generally similar in LGA cases compared to AGA controls. These results provide preliminary evidence that inflammatory processes may be dysregulated in mothers who go on to deliver babies with adverse fetal growth outcomes. Additional study of the role of circulating eicosanoids in pregnancy is warranted.

## Supporting information

S1 STROBE Checklist (PDF)

S1 Appendix Detailed description of liquid chromatography with tandem mass spectrometry. (PDF)

S2 Appendix Detailed description of Bayesian linear mixed effects models. (PDF)

S1 Fig Eicosanoid correlations at mid- and late pregnancy. (PDF)

S2 Fig Longitudinal profiles of fatty acid precursors. (PDF)

S3 Fig Sensitivity analysis stratifying longitudinal profiles of eicosanoids by case status. (PDF)

S1 Table Metabolites included in primary analyses. (DOCX)

S2 Table Metabolites excluded from primary analyses due to LOD. (DOCX)

S3 Table Distribution and variability of analytes across study visits. (DOCX)

S4 Table Sensitivity analysis examining unadjusted associations. (DOCX)

S5 Table Sensitivity analysis excluding women with relevant comorbidities. (DOCX)

S6 Table Sensitivity analysis excluding smokers. (DOCX)

S7 Table Characteristics of participants with non-missing and missing eicosanoid values. (DOCX)

## Abbreviations

AA: arachidonic acid
AGA: appropriate for gestational age
BLM: Bayesian linear mixed effects model
BWH: Brigham and Women’s Hospital
COX: cyclooxygenase
CrI: credible interval
CYP: cytochrome P450
DHA: docosahexaenoic acid
EPA: eicosapentaenoic acid
ICC: intraclass correlation coefficient
LA: linoleic acid
LGA: large for gestational age
LOD: limit of detection
LOX: lipoxygenase
SGA: small for gestational age
