# Supplementary material for: Longitudinal profiles of plasma eicosanoids during pregnancy and size for gestational age at delivery: A nested case-control study
Source: PLoS Med. 2020 Aug 14;17(8):e1003271. doi: 10.1371/journal.pmed.1003271 (PMC7428021; doi:10.1371/journal.pmed.1003271)
Supplement: S1 STROBE Checklist — (PDF) [file pmed.1003271.s001.pdf]

STROBE Statement—Checklist of items that should be included in reports of nested case-control studies

	Item No	Recommendation	Section	Paragraph number in each section
**Title and abstract**	1	(*a*) Indicate the study's design with a commonly used term in the title or the abstract	Title;<br>Abstract;<br>Study population	1;<br>1;<br>1-2
		(*b*) Provide in the abstract an informative and balanced summary of what was done and what was found	Abstract	1-3
**Introduction**				
Background/rationale	2	Explain the scientific background and rationale for the investigation being reported	Introduction	1-2
Objectives	3	State specific objectives, including any prespecified hypotheses	Introduction	3
**Methods**				
Study design	4	Present key elements of study design early in the paper	Introduction;<br>Study population	1;<br>1-2
Setting	5	Describe the setting, locations, and relevant dates, including periods of recruitment, exposure, follow-up, and data collection	Study population	1-2
Participants	6	(*a*) Give the eligibility criteria, and the sources and methods of case ascertainment and control selection. Give the rationale for the choice of cases and controls	Study population	1-2
		(*b*) For matched studies, give matching criteria and the number of controls per case	Study population	2
Variables	7	Clearly define all outcomes, exposures, predictors, potential confounders, and effect modifiers. Give diagnostic criteria, if applicable	Study population;<br>Eicosanoid quantification;<br>Statistical analysis	2;<br>1-2, Fig 1.;<br>2-4
Data sources/ measurement	8*	For each variable of interest, give sources of data and details of methods of assessment (measurement). Describe comparability of assessment methods if there is more than one group	Study population;<br>Eicosanoid quantification;<br>Statistical analysis	2;<br>1-2, Fig 1.;<br>2-4
Bias	9	Describe any efforts to address potential sources of bias	Statistical analysis	4
Study size	10	Explain how the study size was arrived at	Study population	2
Quantitative variables	11	Explain how quantitative variables were handled in the analyses. If applicable, describe which groupings were chosen and why	Eicosanoid quantification;<br>Statistical analysis	2;<br>1-3
Statistical methods	12	(*a*) Describe all statistical methods, including those used to control for confounding	Statistical analysis	1-4
		(*b*) Describe any methods used to examine subgroups and interactions	Statistical analysis	2-4

		(*c*) Explain how missing data were addressed	Statistical analysis	2
		(*d*) If applicable, explain how matching of cases and controls was addressed	Statistical analysis	2
		(*e*) Describe any sensitivity analyses	Statistical analysis	3-4
**Results**				
Participants	13*	(a) Report numbers of individuals at each stage of study—eg numbers potentially eligible, examined for eligibility, confirmed eligible, included in the study, completing follow-up, and analysed	Study population	1-2
		(b) Give reasons for non-participation at each stage	Study population	1-2
		(c) Consider use of a flow diagram	Not included	
Descriptive data	14*	(a) Give characteristics of study participants (eg demographic, clinical, social) and information on exposures and potential confounders	Results; Table 1; Appendix B, Supp. Tables 1-3	1
		(b) Indicate number of participants with missing data for each variable of interest	Study population	2
Outcome data	15*	Report numbers in each exposure category, or summary measures of exposure	Results; Table 1; Appendix B, Supp. Table 3	1, 3
Main results	16	(*a*) Give unadjusted estimates and, if applicable, confounder-adjusted estimates and their precision (eg, 95% confidence interval). Make clear which confounders were adjusted for and why they were included	Figure 4; Appendix B, Supp. Table 4	
		(*b*) Report category boundaries when continuous variables were categorized	Statistical analysis;	2
		(*c*) If relevant, consider translating estimates of relative risk into absolute risk for a meaningful time period	Report percent differences to improve estimate translation	
Other analyses	17	Report other analyses done—eg analyses of subgroups and interactions, and sensitivity analyses	Results	3, 7
**Discussion**				
Key results	18	Summarise key results with reference to study objectives	Discussion	1-6
Limitations	19	Discuss limitations of the study, taking into account sources of potential bias or imprecision. Discuss both direction and magnitude of any potential bias	Strengths and limitations	8-12
Interpretation	20	Give a cautious overall interpretation of results considering objectives, limitations, multiplicity of analyses, results from similar studies, and other relevant evidence	Conclusion	1
Generalisability	21	Discuss the generalisability (external validity) of the study results	Strengths and limitations	8-12
**Other information**				

Funding	22	Give the source of funding and the role of the funders for the present study and, if applicable, for the original study on which the present article is based	Included	

*Give information separately for cases and controls.

Note: An Explanation and Elaboration article discusses each checklist item and gives methodological background and published examples of transparent reporting. The STROBE checklist is best used in conjunction with this article (freely available on the Web sites of PLoS Medicine at http://www.plosmedicine.org/, Annals of Internal Medicine at http://www.annals.org/, and Epidemiology at http://www.epidem.com/). Information on the STROBE Initiative is available at http://www.strobe-statement.org.
