# Supplementary material for: Longitudinal profiles of plasma eicosanoids during pregnancy and size for gestational age at delivery: A nested case-control study
Source: PLoS Med. 2020 Aug 14;17(8):e1003271. doi: 10.1371/journal.pmed.1003271 (PMC7428021; doi:10.1371/journal.pmed.1003271)
Supplement: S1 Appendix — (PDF) [file pmed.1003271.s002.pdf]

S1 Appendix. Detailed description of liquid chromatography with tandem mass spectrometry methods.

**Analytical step**	**Details**
Materials	PGE_2-d4, 8-iso-$PGF_{2\alpha}$-d4, 11,12-DHET-d11, 11,12-EET-d11, 5-iPF2a-VI-d4, AA-d8, d4-LTB4 as well as all non-deuterated pure chemicals from all eicosanoid and fatty acid analytes were purchased from Cayman Chemical (Ann Arbor, MI, USA). All solvents used for SPE extraction or liquid chromatography with tandem mass spectrometry (LC-MS/MS) are HPLC grade or higher from Sigma (St Louis, MO, USA).
Sample preparation	Prior to analysis, plasma samples were thawed, spiked with 10 ng PGE_2-d4, 8-iso-$PGF_{2\alpha}$-d4, 11,12-DHET-d11, 11,12-EET-d11, 5-iPF2a-VI-d4, AA-d8, d4-LTB4 as internal standards, and kept on ice during sample preparation. 1 mL of plasma was subjected to solid-phase extraction using 3 mL HLB-prime SPE columns (Waters Corp, Billerica, MA, USA). SPE columns were prewashed with 3 mL of butyl acetate, 3 mL acetone and 6 mL water. Plasma aliquots were acidified prior to deposition on the column with 1 mL of 1 % acetic acid and 5 % methanol in 100 X 13 mm glass tubes. Prewashed columns and acidified samples were loaded into the Rapidtrace$^+$ automated SPE workstation (Biotage LLC., Charlotte, NC, USA) 10 samples at a time.<br><br>The Rapidtrace executed the following protocol: 1) load sample (2.2 mL); 2) rinse column with 5% methanol / 0.1 % acetic acid (1 mL); 3) dry with nitrogen stream for 30 seconds; and 4) collect with butyl acetate (2.5 mL). This process occurred in batches of 10 randomly selected samples. After every ~30-40 samples a new cartridge was extracted with 1 mL of the acidification mixture as a blank. The Rapidtrace was flushed with butyl acetate and methanol in addition to acidic and basic water solution after every day of processing to minimize contamination. Samples were evaporated in a centrifugal vacuum evaporator maintained at 40 ºC and reconstituted with 50 µL of a 33 % ethanol / 0.5 % acetic acid solution. After the above preparation, samples were kept at -80 ºC in glass auto sampler vials prior to analysis by LC-MS/MS.
LC-MS/MS fatty acid and eicosanoid analysis	Fatty acids and eicosanoid levels were determined in extracted plasma samples by LC-MS/MS as previously described [1]. Online liquid chromatography and electrospray ionization tandem mass spectrometry of extracted plasma samples were performed on an UltiMate 3000 RS HPLC system and Quantiva mass spectrometer (Thermo Fisher Scientific, Waltham, MA, USA). Separations were achieved using a HALO C18 column (2.0 µm, 100 x 2.1 mm, Advanced Materials Technology, Wilmington, DE, USA), which was held at 50°C. The flow rate was 500 µL/min. Mobile phase A was 2 mM acetic acid in 97:3 water / n-propanol. Mobile phase B was acetonitrile. Gradient elution was used, and mobile phase B was varied as follows: 20% B from 0 min to 0.5 min, linear ramp to 50% B at 6 min, ramp with curve 8 to 97% B at 12.5 min, held at 97% B to 13.5 min. The column was re-equilibrated with 725 µL starting mobile phase prior to initiating the next injection. Injection volume was 10 µL. All analytes were monitored as parent ion-product ion, mass /

	charge pairs with specific retention times as negative ions in a selected reaction monitoring experiment.
MS data analysis	Peaks identified as fatty acids or eicosanoids were integrated and normalized to their assigned deuterated standards. The normalized peak areas were converted to concentrations using quantitation curves generated together with the standard samples. Samples were analyzed without prior knowledge of outcome data.
Quality control	The accuracy of analytical runs was considered acceptable if the peak area of each internal standard was between 75%-120% of the true mean value. There were 12 samples that failed this criterion, which was attributed to a faulty injection from insufficient sample volume or insufficient solid phase extraction recovery.

References

1. Newman, J.W., T. Watanabe, and B.D. Hammock, *The simultaneous quantification of cytochrome P450 dependent linoleate and arachidonate metabolites in urine by HPLC-MS/MS.* J Lipid Res, 2002. **43**(9): p. 1563-78.
