# Supplementary material for: Longitudinal profiles of plasma eicosanoids during pregnancy and size for gestational age at delivery: A nested case-control study
Source: PLoS Med. 2020 Aug 14;17(8):e1003271. doi: 10.1371/journal.pmed.1003271 (PMC7428021; doi:10.1371/journal.pmed.1003271)
Supplement: S2 Appendix — (PDF) [file pmed.1003271.s003.pdf]

S2 Appendix. Detailed description of Bayesian linear mixed model methods.

Model process	Details
Model specification settings	Since specific prior information for eicosanoid distributions was not available, relatively non-informative priors for standardized model parameters were specified. This included a gaussian distribution for population-level main effects (mean = 0, standard deviation = 1), and a student's t-distribution for population-level standard deviation, residual standard deviation, and variance of smooth terms (mean = 0, variance = 10). A total of 12,000 iterations and 6,000 warm-up (burn-in) iterations over 4 Markov chains (MC) were used for each Bayesian linear mixed model (BLM). The model fit of each BLM was examined visually (MC mixing in trace plots, posterior predictive distribution plots) and empirically (scale reduction factors [Rhat] near 1.0, effective degrees of freedom). Additional details on BLM settings can be found in the R code provided below.

R Code - unadjusted model

```
# GLOBAL BAYES REGRESSION SETTINGS ####
## Family parameters ##
# family = student # robust linear regress (less outlier influenced) of student t
distribution
# student family requires nu parameter (set to default)

## Prior classes ##
# "b"           : population-level effects
# "Intercept"   : population-level intercept
# "sd" : population-level standard deviation
# "sigma": population-level model error term, residual SD
# "sds": population-level standard deviation of coefficients, used for spline
# "nu": degrees of freedom of students-t distribution. By default, nu has

#FAMILY
family_brm = student #student=robust linear regression
   #By setting family=student, automatically induces add'n prior of
prior(gamma(2,0.1),class=nu)

#PRIORS
prior_formula <- c(prior(normal(0,1), class = b),
                   prior(student_t(3, 0, 10), class=Intercept),
                   prior(student_t(5, 0, 10), class = sd),
                   prior(student_t(5, 0, 10), class = sigma),
                   prior(student_t(5, 0, 10), class = sds),
                   prior(gamma(2,0.1),class=nu))
#Iterations and control
  iter_brm <- 3000 #number of iterations per chain (including warmup, default is
2000)
  warmup_brm <- 1500 #warmup iterations
  control_brm <- list(adapt_delta=.999, max_treedepth = 22)
```

	#MCMC stuff (default)<br>chains_brm <- 4 #number of MC chains. default is 4<br>cores_brm <- 2 #number of cores. May be able to increase?<br>seed_brm <- 5645 #set seed for reproducibility<br><br>##############<br># BRM MODEL - pge2 - Compiling of C++ model ####<br>pge2_formula <- pge2~ s(ga) + growcat + (1|id) #pge2 formula<br>pge2_brm1 <- brm(data = df,<br>family = family_brm,<br>formula = pge2_formula,<br>prior = prior_formula,<br>iter = iter_brm, warmup = warmup_brm,<br>control = control_brm,<br>chains = chains_brm, cores = cores_brm, seed=seed_brm)<br><br># This compiled model is then used for subsequent independent biomarker models
