# Supplementary material for: Longitudinal profiles of plasma eicosanoids during pregnancy and size for gestational age at delivery: A nested case-control study
Source: PLoS Med. 2020 Aug 14;17(8):e1003271. doi: 10.1371/journal.pmed.1003271 (PMC7428021; doi:10.1371/journal.pmed.1003271)

S1 Fig. Eicosanoid correlations at mid and late pregnancy.
Eicosanoids are grouped by biosynthetic pathways.

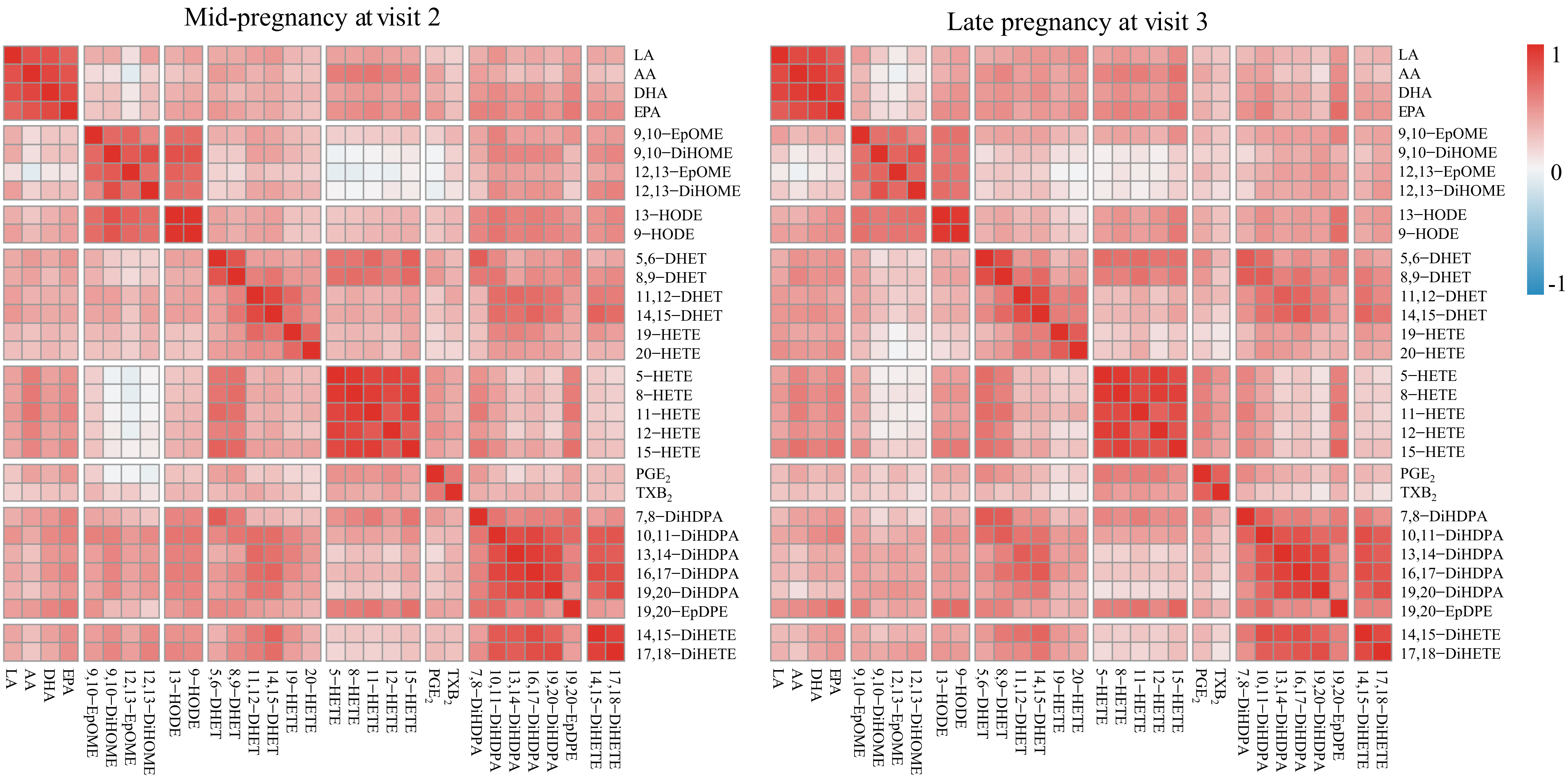

Supplement: S1 Fig — (PDF) [file pmed.1003271.s004.pdf]
