# Supplementary material for: Longitudinal profiles of plasma eicosanoids during pregnancy and size for gestational age at delivery: A nested case-control study
Source: PLoS Med. 2020 Aug 14;17(8):e1003271. doi: 10.1371/journal.pmed.1003271 (PMC7428021; doi:10.1371/journal.pmed.1003271)

S2 Fig. Longitudinal profiles of fatty acid precursors.
Profiles estimated by Bayesian linear mixed models for linoleic acid (LA), arachidonic acid (AA), docosahexaenoic (DHA) or eicosapentaenoic acid (EPA).

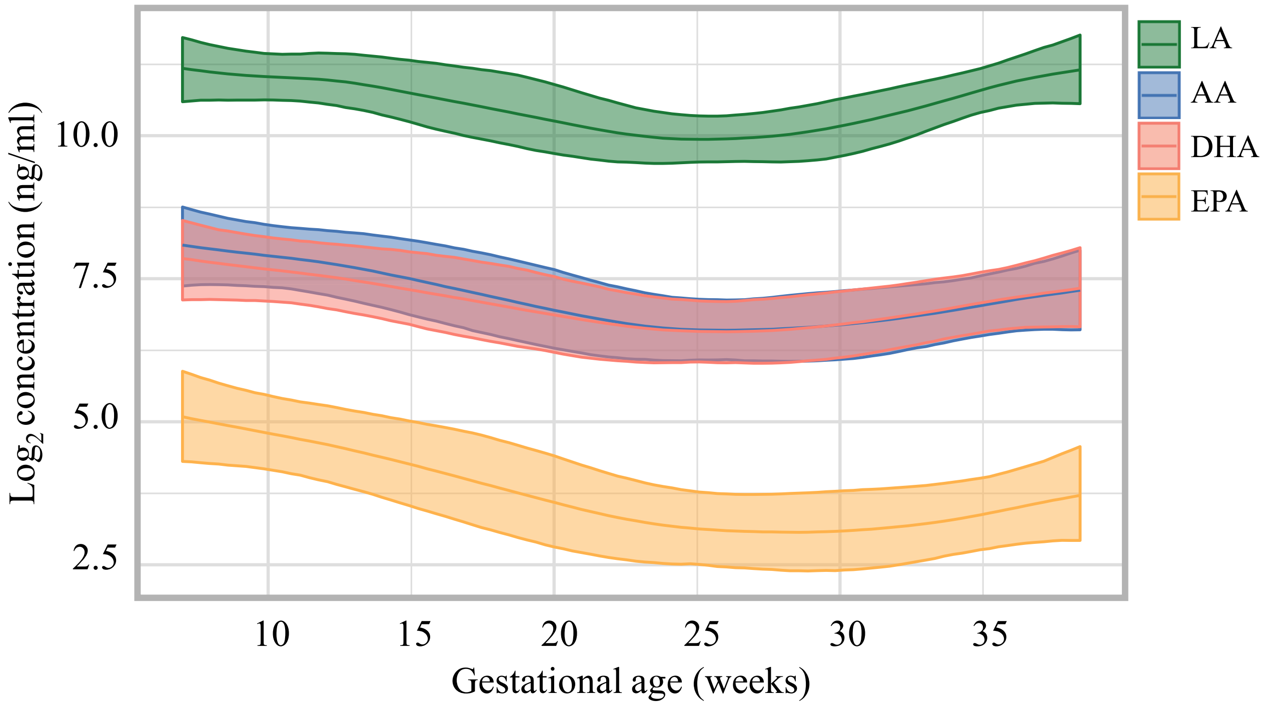

Supplement: S2 Fig — (PDF) [file pmed.1003271.s005.pdf]
