# Supplementary material for: Longitudinal profiles of plasma eicosanoids during pregnancy and size for gestational age at delivery: A nested case-control study
Source: PLoS Med. 2020 Aug 14;17(8):e1003271. doi: 10.1371/journal.pmed.1003271 (PMC7428021; doi:10.1371/journal.pmed.1003271)
Supplement: S3 Fig — (PDF) [file pmed.1003271.s006.pdf]

S3 Fig. Longitudinal profiles of eicosanoids stratified by case status. Profiles estimated by Bayesian linear mixed models separately for mothers of small for gestational age (SGA), controls, and large for gestational age (LGA) births.

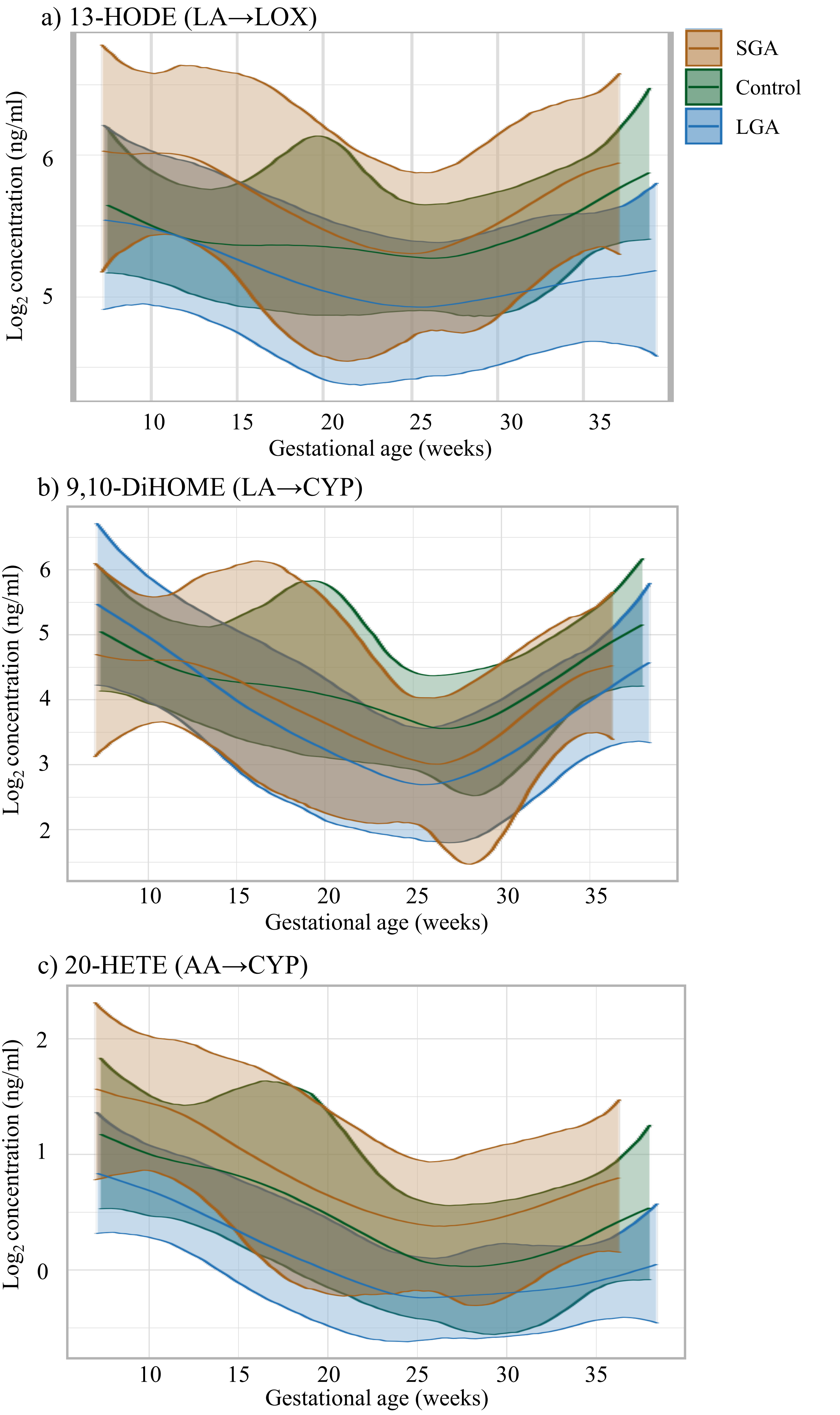

d) 11-HETE (AA→LOX)

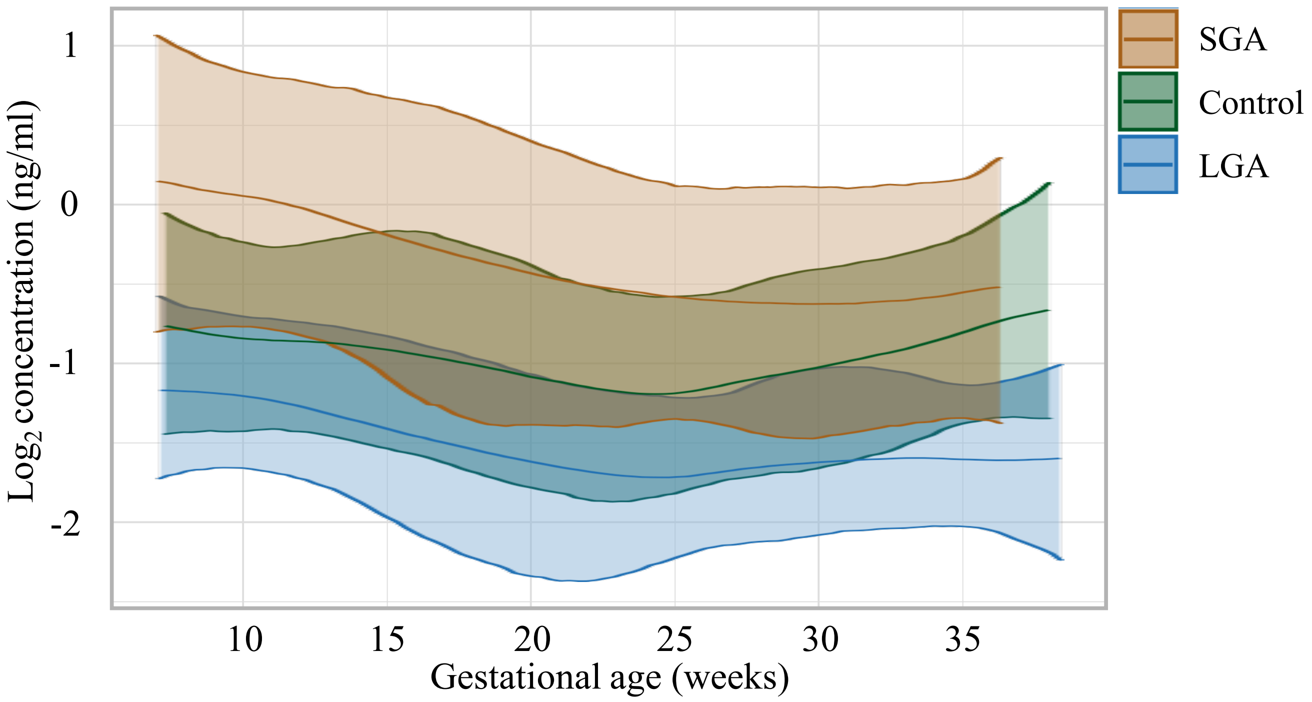

e) PGE$_2$ (AA→COX)

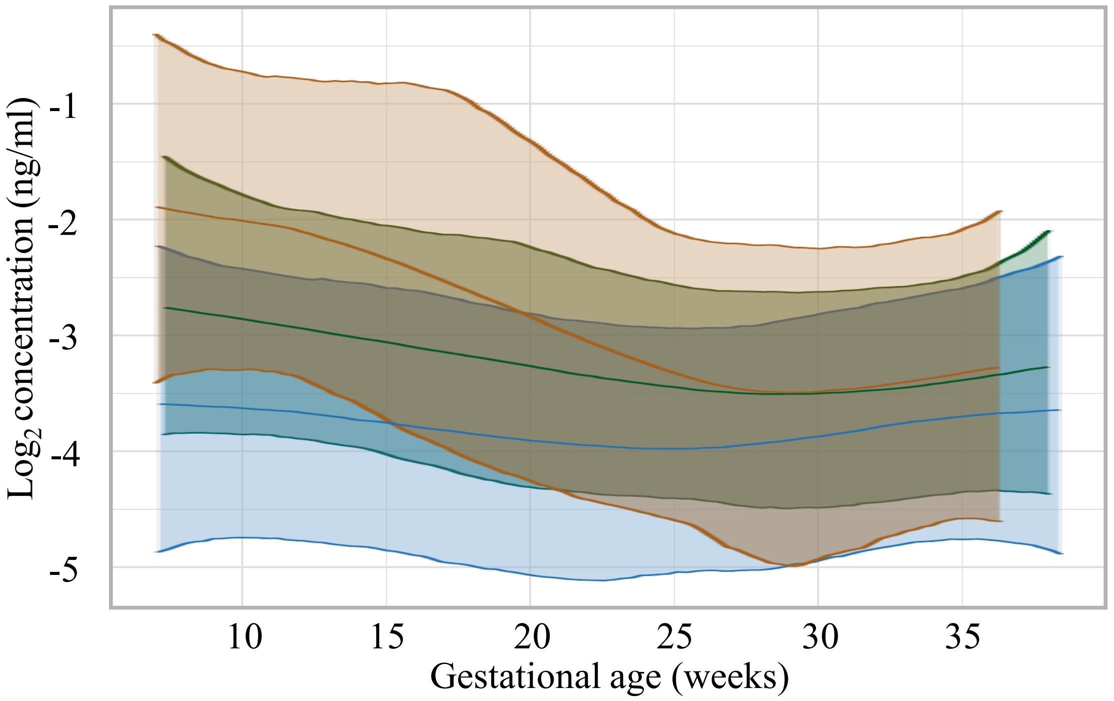

f) 17,18-DiHETE (EPA→CYP)

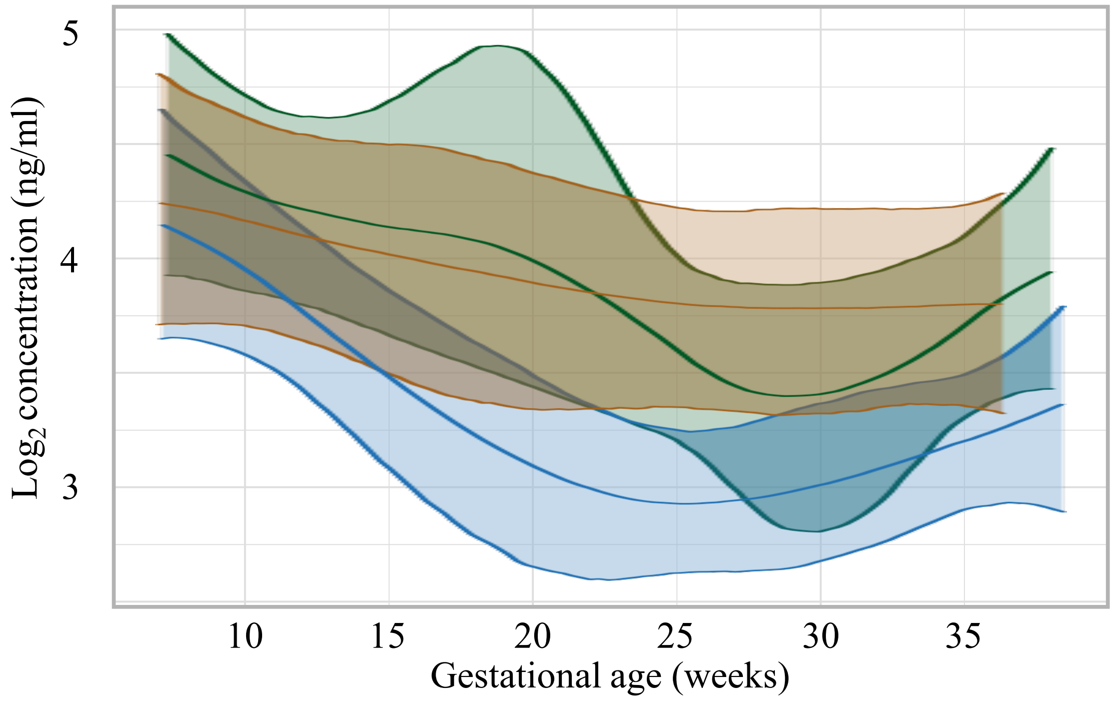

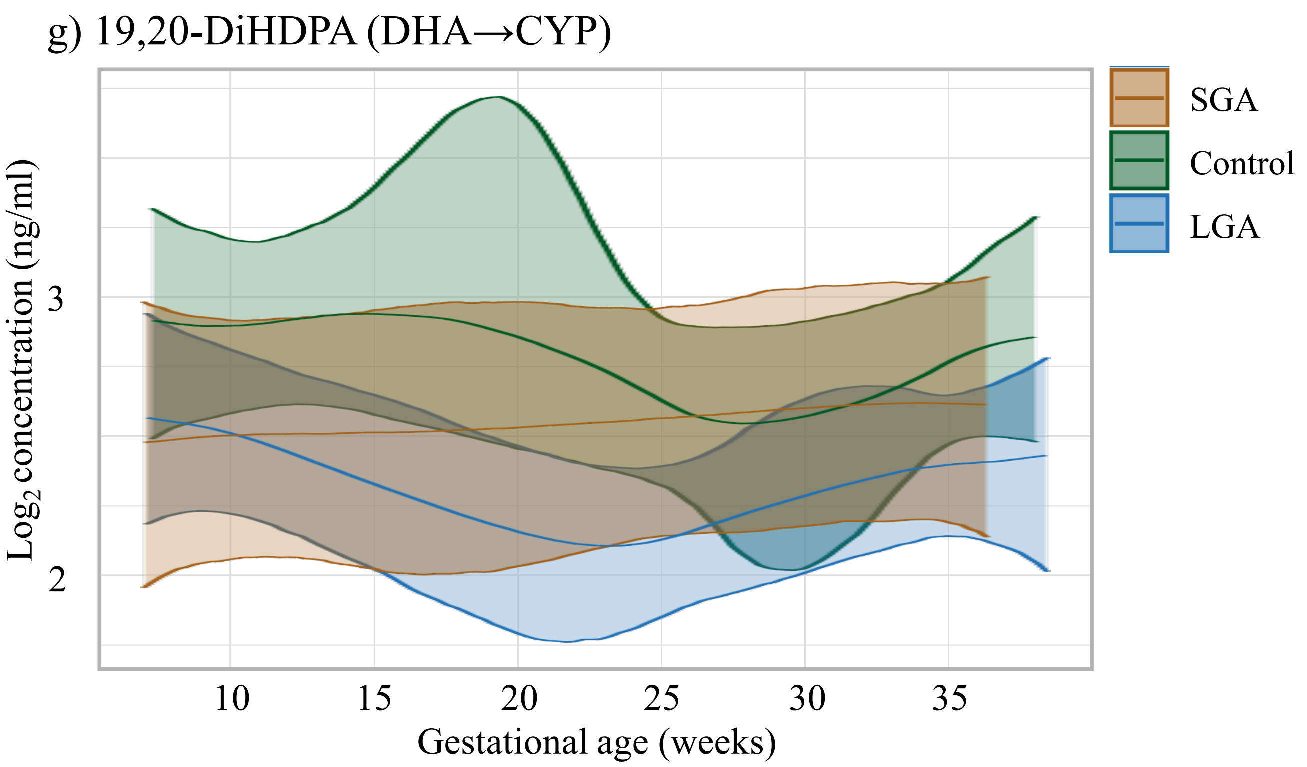
g) 19,20-DiHDPA (DHA→CYP)
Log2 concentration (ng/ml)
3
2
10
15
20
25
30
35
Gestational age (weeks)
SGA
Control
LGA
