# Supplementary material for: Longitudinal profiles of plasma eicosanoids during pregnancy and size for gestational age at delivery: A nested case-control study
Source: PLoS Med. 2020 Aug 14;17(8):e1003271. doi: 10.1371/journal.pmed.1003271 (PMC7428021; doi:10.1371/journal.pmed.1003271)
Supplement: S1 Table — (DOCX) [file pmed.1003271.s007.docx]

**S1 Table. Metabolites with ≥50% of samples above LOD and included in primary analyses.**

|  |  |  |  | LOD | >LOD |
| --- | --- | --- | --- | --- | --- |
| Grouping^a^ | | Full name | Abbreviation | (ng/ml) | (%) |
| Fatty | acid | Linoleic acid | LA | 3.000 | 100.0% |
|  |  | Arachidonic acid | AA | 3.000 | 100.0% |
|  |  | Docosahexaenoic acid | DHA | 3.000 | 99.6% |
|  |  | Eicosapentaenoic acid | EPA | 3.000 | 67.8% |
| Pathway | |  |  |  |  |
| Fatty acid | Enzyme | Eicosanoid |  |  |  |
| LA | CYP | 9,10-epoxy-octadecenoic acid | 9,10-EpOME | 0.500 | 100.0% |
|  |  | 9,10-dihydroxy-octadecenoic acid | 9,10-DiHOME | 1.000 | 88.6% |
|  |  | 12,13-epoxy-octadecenoic acid | 12,13-EpOME | 0.500 | 100.0% |
|  |  | 12,13-dihydroxy-octadecenoic acid | 12,13-DiHOME | 1.000 | 68.6% |
|  | LOX | 13-hydroxy-octadecadienoic acid | 13-HODE | 2.500 | 100.0% |
|  |  | 9-hydroxy-octadecadienoic acid | 9-HODE | 2.500 | 100.0% |
| AA | CYP | 5,6-dihydroxy-eicosatrienoic acid | 5,6-DHET | 0.100 | 98.0% |
|  |  | 8,9-dihydroxy-eicosatrienoic acid | 8,9-DHET | 0.100 | 98.0% |
|  |  | 11,12-dihydroxy-eicosatrienoic acid | 11,12-DHET | 0.100 | 99.6% |
|  |  | 14,15-dihydroxy-eicosatrienoic acid | 14,15-DHET | 0.100 | 99.6% |
|  |  | 19-hydroxy-eicosatetraenoic acid | 19-HETE | 0.500 | 85.3% |
|  |  | 20-hydroxy-eicosatetraenoic acid | 20-HETE | 0.500 | 89.0% |
|  | LOX | 5-hydroxy-eicosatetraenoic acid | 5-HETE | 0.100 | 68.2% |
|  |  | 8-hydroxy-eicosatetraenoic acid | 8-HETE | 0.100 | 84.5% |
|  |  | 11-hydroxy-eicosatetraenoic acid | 11-HETE | 0.100 | 99.2% |
|  |  | 12-hydroxy-eicosatetraenoic acid | 12-HETE | 0.613 | 51.8% |
|  |  | 15-hydroxy-eicosatetraenoic acid | 15-HETE | 0.100 | 100.0% |
|  | COX | Prostaglandin E2 | PGE_2_ | 0.025 | 59.2% |
|  |  | Thromboxone B2 | TXB_2_ | 0.125 | 80.0% |
| DHA | CYP | 7,8-dihydroxy-docosapentaenoic acid | 7,8-DiHDPA | 0.100 | 79.6% |
|  |  | 10,11-dihydroxy-docosapentaenoic acid | 10,11-DiHDPA | 0.100 | 93.9% |
|  |  | 13,14-dihydroxy-docosapentaenoic acid | 13,14-DiHDPA | 0.100 | 99.2% |
|  |  | 16,17-dihydroxy-docosapentaenoic acid | 16,17-DiHDPA | 0.100 | 98.8% |
|  |  | 19,20-dihydroxy-docosapentaenoic acid | 19,20-DiHDPA | 0.100 | 99.6% |
|  |  | 19,20-epoxy-docosapentaenoic acid | 19,20-EpDPE | 0.200 | 98.4% |
| EPA | CYP | 14,15-dihydroxy-eicosatetraenoic acid | 14,15-DiHETE | 0.100 | 97.1% |
|  |  | 17,18-dihydroxy-eicosatetraenoic acid | 17,18-DiHETE | 0.100 | 99.6% |

^a^ Abbreviations: COX, cyclooxygenase; CYP, cytochrome P450; LOX, lipoxygenase.
