# Supplementary material for: Longitudinal profiles of plasma eicosanoids during pregnancy and size for gestational age at delivery: A nested case-control study
Source: PLoS Med. 2020 Aug 14;17(8):e1003271. doi: 10.1371/journal.pmed.1003271 (PMC7428021; doi:10.1371/journal.pmed.1003271)
Supplement: S2 Table — (DOCX) [file pmed.1003271.s008.docx]

**S2 Table. Metabolites with <50% samples above LOD and subsequently dropped from further analyses.**

|  |  |  | |  | | LOD | | >LOD | |  |
| --- | --- | --- | --- | --- | --- | --- | --- | --- | --- | --- |
| Grouping^a^ | | Full name | | Abbreviation | | (ng/ml) | | (%) | |  |
| Pathway | |  | |  | |  | |  | |  |
| Fatty acid | Enzyme | | Eicosanoid | |  | |  | |  | |
| AA | CYP | 11,12-epoxy-eicosatrienoic acid | | 11,12-EET | | 0.200 | | 3% | |  |
|  |  | 14,15-epoxy-eicosatrienoic acid | | 14,15-EET | | 0.200 | | 43% | |  |
|  |  | 17,18-epoxy-eicosatrienoic acid | | 17,18-EET | | 0.100 | | 3% | |  |
|  |  | 20-carboxy-leukotriene-B4 | | 20-carboxy-LTB4 | | 0.025 | | 4% | |  |
|  |  | 5,6-epoxy-eicosatrienoic acid | | 5,6-EET | | 16.000 | | non-detect | |  |
|  |  | 8,9-epoxy-eicosatrienoic acid | | 8,9-EET | | 0.200 | | non-detect | |  |
|  |  | 20-hydroxy prostaglandin E_2_ | | 20-OH-PGE2 | | 0.025 | | 2% | |  |
|  | LOX | Lipoxin A4 | | Lipoxin A_4_ | | 0.025 | | 18% | |  |
|  |  | Lipoxin B4 | | Lipoxin B_4_ | | 0.025 | | 21% | |  |
|  |  | Leukotriene B4 | | LTB_4_ | | 0.025 | | 2% | |  |
|  | COX | Prostaglandin B2 | | PGB_2_ | | 2.000 | | 2% | |  |
|  |  | Prostaglandin D2 | | PGD_2_ | | 0.125 | | 38% | |  |
|  |  | Prostaglandin D3 | | PGD_3_ | | 0.125 | | 5% | |  |
|  |  | Prostaglandin F2α | | PGF_2α_ | | 0.050 | | 40% | |  |
|  |  | 6-keto Prostaglandin F1α | | 6ketoPGF1a | | 0.100 | | non-detect | |  |
| DHA | CYP | 10,11-epoxy-docosapentaenoic acid | | 10,11-EpDPA | | 0.200 | | 15% | |  |
|  |  | 13,14-epoxy-docosapentaenoic acid | | 13,14-EpDPA | | 0.200 | | non-detect | |  |
|  |  | 16,17-epoxy-docosapentaenoic acid | | 16,17-EpDPA | | 0.200 | | non-detect | |  |
|  |  | 7,8-epoxy-docosapentaenoic acid | | 7,8-EpDPA | | 0.200 | | 31% | |  |
|  | LOX | Resolvin D1 | | Resolvin_D1 | | 0.125 | | 1% | |  |
|  |  | Resolvin D2 | | Resolvin_D2 | | 0.500 | | non-detect | |  |
|  |  | Resolvin D3 | | Resolvin_D3 | | 0.050 | | non-detect | |  |
|  |  | Resolvin D4 | | Resolvin_D4 | | 1.000 | | non-detect | |  |
|  |  | 17-hydroxy-docosahexaenoic acid | | 17-HDHA | | 0.025 | | 38% | |  |
| EPA | CYP | 11,12-dihydroxy-eicosatetraenoic acid | | 11,12-DiHETE | | 0.100 | | non-detect | |  |
|  |  | 11,12-epoxy-eicosatetraenoic acid | | 11,12-EpETE | | 0.200 | | non-detect | |  |
|  |  | 14,15-epoxy-eicosatetraenoic acid | | 14,15-EpETE | | 0.200 | | 2% | |  |
|  |  | 17,18-epoxy-eicosatetraenoic acid | | 17,18-EpETE | | 0.100 | | 2% | |  |
|  | COX | Prostaglandin E3 | | PGE_3_ | | 0.025 | | 8% | |  |
|  |  | Resolvin E1 | | Resolvin_E1 | | 0.025 | | non-detect | |  |

^a^ Abbreviations: AA, arachidonic acid; COX, cyclooxygenase; CYP, cytochrome P450; DHA, docosahexaenoic acid; EPA, eicosapentaenoic acid; LA, linoleic acid; LOX, lipoxygenase.
