# Supplementary material for: Longitudinal profiles of plasma eicosanoids during pregnancy and size for gestational age at delivery: A nested case-control study
Source: PLoS Med. 2020 Aug 14;17(8):e1003271. doi: 10.1371/journal.pmed.1003271 (PMC7428021; doi:10.1371/journal.pmed.1003271)
Supplement: S3 Table — (DOCX) [file pmed.1003271.s009.docx]

**S3 Table. Maternal plasma fatty acid and eicosanoid distributions (ng/ml) and variability across study visits.^a^**

|  |  |  | median (IQR) | | | ICC |
| --- | --- | --- | --- | --- | --- | --- |
| Grouping^b^ | | Biomarker | Visit 1 (n=80) | Visit 2 (n=86) | Visit 3 (n=79) | ρ (95% CI) |
| Fatty acid | | LA | 1,937 (1,042, 3,738) | 987 (507, 1,560) | 1,831 (976, 3,283) | 0.18 (0.07, 0.36) |
|  |  | AA | 256 (92, 505) | 70 (40, 221) | 152 (53, 308) | 0.33 (0.20, 0.48) |
|  |  | DHA | 193 (86, 412) | 78 (31, 168) | 125 (59, 236) | 0.32 (0.19, 0.47) |
|  |  | EPA | 30.9 (5.4, 55.1) | 5.2 (2.1, 19.0) | 7.3 (2.1, 30.7) | 0.36 (0.23, 0.50) |
| Pathway | |  |  |  |  |  |
| Fatty acid | Enzyme | Eicosanoid |  |  |  |  |
| LA | CYP | 9,10-EpOME | 16.2 (10.1, 22.5) | 9.1 (5.3, 14.0) | 14.2 (8.1, 22.0) | 0.06 (0.00, 0.38) |
|  |  | 9,10-DiHOME | 27.0 (14.8, 42.2) | 7.2 (0.7, 26.5) | 23.1 (11.3, 33.1) | 0.02 (0.00, 0.90) |
|  |  | 12,13-EpOME | 32.1 (21.7, 52.9) | 20.0 (11.4, 38.0) | 27.2 (16.9, 48.3) | 0.18 (0.07, 0.36) |
|  |  | 12,13-DiHOME | 36.4 (13.0, 74.1) | 0.7 (0.7, 29.4) | 29.2 (11.8, 49.8) | 0.03 (0.00, 0.57) |
|  | LOX | 13-HODE | 40.2 (28.4, 59.7) | 29.0 (15.4, 48.8) | 38.5 (24.9, 60.0) | 0.17 (0.07, 0.35) |
|  |  | 9-HODE | 36.8 (22.6, 53.4) | 24.2 (13.3, 41.8) | 32.3 (21.3, 52.6) | 0.21 (0.09, 0.38) |
| AA | CYP | 5,6-DHET | 1.1 (0.8, 2.0) | 1.0 (0.6, 1.5) | 0.9 (0.7, 1.5) | 0.46 (0.34, 0.59) |
|  |  | 8,9-DHET | 1.2 (0.9, 1.8) | 1.0 (0.7, 1.4) | 1.1 (0.8, 1.8) | 0.44 (0.31, 0.57) |
|  |  | 11,12-DHET | 2.0 (1.6, 2.6) | 1.3 (1.1, 1.7) | 1.7 (1.4, 2.5) | 0.22 (0.10, 0.38) |
|  |  | 14,15-DHET | 2.8 (2.2, 3.5) | 1.9 (1.5, 2.5) | 2.5 (2.0, 3.5) | 0.29 (0.17, 0.44) |
|  |  | 19-HETE | 1.4 (1.0, 2.0) | 0.9 (0.4, 1.0) | 1.0 (0.9, 1.6) | 0.20 (0.09, 0.36) |
|  |  | 20-HETE | 2.0 (1.5, 3.0) | 1.0 (0.6, 1.6) | 1.2 (0.9, 1.8) | 0.16 (0.06, 0.34) |
|  | LOX | 5-HETE | 0.3 (0.1, 0.6) | 0.1 (0.1, 0.4) | 0.2 (0.1, 0.4) | 0.39 (0.26, 0.53) |
|  |  | 8-HETE | 0.3 (0.2, 0.8) | 0.2 (0.1, 0.5) | 0.3 (0.1, 0.6) | 0.40 (0.27, 0.54) |
|  |  | 11-HETE | 0.6 (0.4, 0.9) | 0.3 (0.2, 0.7) | 0.5 (0.3, 0.8) | 0.38 (0.26, 0.52) |
|  |  | 12-HETE | 1.0 (0.4, 2.7) | 0.4 (0.4, 1.4) | 0.6 (0.4, 2.1) | 0.38 (0.25, 0.52) |
|  |  | 15-HETE | 0.9 (0.7, 1.5) | 0.5 (0.4, 1.0) | 0.7 (0.5, 1.1) | 0.36 (0.23, 0.50) |
|  | COX | PGE_2_ | 0.15 (0.02, 0.55) | 0.02 (0.02, 0.22) | 0.08 (0.02, 0.36) | 0.43 (0.31, 0.57) |
|  |  | TXB_2_ | 0.9 (0.4, 1.8) | 0.3 (0.1, 1.1) | 0.6 (0.3, 1.6) | 0.32 (0.19, 0.47) |
| DHA | CYP | 7,8-DiHDPA | 0.4 (0.2, 0.5) | 0.4 (0.2, 0.4) | 0.4 (0.3, 0.7) | 0.50 (0.37, 0.62) |
|  |  | 10,11-DiHDPA | 0.3 (0.2, 0.4) | 0.3 (0.2, 0.3) | 0.3 (0.2, 0.4) | 0.47 (0.35, 0.60) |
|  |  | 13,14-DiHDPA | 0.5 (0.4, 0.8) | 0.4 (0.3, 0.6) | 0.5 (0.4, 0.7) | 0.37 (0.24, 0.51) |
|  |  | 16,17-DiHDPA | 0.8 (0.5, 1.1) | 0.6 (0.4, 0.9) | 0.7 (0.5, 1.0) | 0.39 (0.26, 0.53) |
|  |  | 19,20-DiHDPA | 5.9 (4.2, 8.2) | 5.1 (3.6, 7.0) | 5.6 (4.2, 7.7) | 0.39 (0.26, 0.53) |
|  |  | 19,20-EpDPE | 3.2 (2.1, 4.4) | 2.2 (1.3, 3.8) | 2.7 (1.8, 5.0) | 0.28 (0.16, 0.44) |
| EPA | CYP | 14,15-DiHETE | 1.1 (0.7, 1.8) | 0.6 (0.3, 1.1) | 0.7 (0.5, 1.3) | 0.38 (0.26, 0.52) |
|  |  | 17,18-DiHETE | 15.5 (9.7, 23.4) | 9.1 (6.6, 14.3) | 10.4 (7.5, 16.0) | 0.35 (0.23, 0.50) |

^a^ Median gestational age at visits 1, 2, and 3 were 11.1 weeks, 25.6 weeks, and 35 weeks, respectively with a sample size of 89 participant.

^b^ Eicosanoids are grouped biosynthetic pathway, including 1) fatty acid precursor and 2) enzyme. Abbreviations for fatty acids and enzymes include: LA, linoleic acid; AA, arachidonic acid; DHA, docosahexaenoic acid; EPA, eicosapentaenoic acid; COX, cyclooxygenase; CYP, cytochrome P450; LOX, lipoxygenase. Additional abbreviations include: ICC, intraclass correlation coefficient; IQR, interquartile range values of 25^th^ and 75^th^ percentiles.
