# Supplementary material for: Longitudinal profiles of plasma eicosanoids during pregnancy and size for gestational age at delivery: A nested case-control study
Source: PLoS Med. 2020 Aug 14;17(8):e1003271. doi: 10.1371/journal.pmed.1003271 (PMC7428021; doi:10.1371/journal.pmed.1003271)
Supplement: S4 Table — (DOCX) [file pmed.1003271.s010.docx]

**S4 Table.** **Sensitivity analysis examining unadjusted associations.**Percent differences and 95% credible intervals (CrI) in the concentrations of maternal plasma biomarkers in small for gestational age (SGA) and large for gestational age (LGA) cases compared to adequate growth controls (referent).^a,b^

|  |  |  | Percent (%) difference | |
| --- | --- | --- | --- | --- |
| Grouping^c^ | | Metabolite | SGA | LGA |
| Fatty | acid | LA | 39.9 (3.8, 84) | -33 (-50.3, -11.6) |
|  |  | AA | 59.5 (5.5, 129) | -38.8 (-59.6, -11.8) |
|  |  | DHA | 47.5 (-1.6, 113.2) | -37.2 (-59.3, -6.9) |
|  |  | EPA | 88.4 (11.9, 197.3) | -36.6 (-62.7, 0.8) |
| Pathway | |  |  |  |
| Fatty acid | Enzyme | Eicosanoid |  |  |
| LA | CYP | 9,10-EpOME | 4.8 (-20, 35.5) | -1.6 (-25, 27.8) |
|  |  | 9,10-DiHOME | 0.1 (-35.7, 48.8) | 1.4 (-35.8, 51.8) |
|  |  | 12,13-EpOME | -3 (-28.7, 29.4) | 32 (-3.2, 78) |
|  |  | 12,13-DiHOME | 40.3 (-23.8, 138.2) | 13.6 (-39, 96.3) |
|  | LOX | 13-HODE | 24.1 (-4.6, 58.7) | 0.9 (-23, 29.2) |
|  |  | 9-HODE | 20.9 (-8.2, 56.5) | 5.3 (-20.9, 36.6) |
| AA | CYP | 5,6-DHET | 39.3 (3, 85.8) | 0.1 (-25.7, 32.9) |
|  |  | 8,9-DHET | 33.2 (4.9, 68.3) | -3.2 (-23.8, 22.5) |
|  |  | 11,12-DHET | 14.2 (-2, 32) | 5.5 (-9.1, 22.1) |
|  |  | 14,15-DHET | 20.9 (3.9, 39.3) | -7.8 (-20.5, 7.4) |
|  |  | 19-HETE | 18.7 (-4.3, 45.5) | 1.5 (-18, 24.9) |
|  |  | 20-HETE | 27.3 (-2, 62.1) | -0.4 (-23.4, 26.9) |
|  | LOX | 5-HETE | 53 (-3.1, 128.8) | -17.4 (-47.2, 22.7) |
|  |  | 8-HETE | 55.3 (-0.6, 130.5) | -9 (-41.8, 34.7) |
|  |  | 11-HETE | 38.6 (-0.4, 88.9) | -10.5 (-35.9, 21.8) |
|  |  | 12-HETE | 57.6 (9.1, 120.3) | -14.5 (-40.8, 19.6) |
|  |  | 15-HETE | 42.6 (7.6, 85.4) | -8 (-31.1, 19.2) |
|  | COX | PGE_2_ | 34.9 (-32.8, 139.3) | -6.1 (-53.3, 68) |
|  |  | TXB_2_ | -14.1 (-48.4, 36.2) | 0.7 (-40, 58.4) |
| DHA | CYP | 7,8-DiHDPA | 19.4 (-21.8, 73.7) | -1.5 (-35.3, 42.4) |
|  |  | 10,11-DiHDPA | 26 (-5, 63.1) | -3.4 (-27.2, 25.4) |
|  |  | 13,14-DiHDPA | 7.3 (-13.8, 31.6) | 0.1 (-18.8, 22) |
|  |  | 16,17-DiHDPA | 7.2 (-13.4, 31.4) | -7.8 (-25.4, 13.9) |
|  |  | 19,20-DiHDPA | 2.5 (-17.4, 25.8) | -7.3 (-25.6, 13.2) |
|  |  | 19,20-EpDPE | 12.8 (-15.9, 48.8) | -9.5 (-32.9, 19.1) |
| EPA | CYP | 14,15-DiHETE | 27 (-4.9, 66.8) | -18.1 (-39.1, 7.3) |
|  |  | 17,18-DiHETE | 16.4 (-10, 47.6) | -14.7 (-34.1, 8.6) |

^a^ Results generated by Bayesian linear mixed models that regressed plasma biomarkers on fetal growth outcomes, which included participant-specific random intercepts and penalized splines on gestational age. Total sample size for models was 89 participants.

^b^ Biomarker concentrations were log2-transformed prior to modeling and standardized population means of BLMs were estimated to calculate percent differences between fetal growth outcome categories.

^c^ Abbreviations: AA, arachidonic acid; COX, cyclooxygenase; CYP, cytochrome P450; DHA, docosahexaenoic acid; EPA, eicosapentaenoic acid; LA, linoleic acid; LOX, lipoxygenase.
