# Supplementary material for: Longitudinal profiles of plasma eicosanoids during pregnancy and size for gestational age at delivery: A nested case-control study
Source: PLoS Med. 2020 Aug 14;17(8):e1003271. doi: 10.1371/journal.pmed.1003271 (PMC7428021; doi:10.1371/journal.pmed.1003271)
Supplement: S5 Table — (DOCX) [file pmed.1003271.s011.docx]

**S5 Table. Sensitivity analysis excluding women with relevant comorbidities.**Percent differences and 95% credible intervals (CrI) in plasma biomarkers from models^a,b^ restricted to women with no relevant comorbidity diagnosis (n=76).^c^

|  |  |  | Percent (%) difference | |
| --- | --- | --- | --- | --- |
| Grouping | | Metabolite | SGA | LGA |
| Fatty | acid | LA | 37.6 (-3.2, 88.5) | -29.3 (-49, -5.5) |
|  |  | AA | 55.7 (-5.9, 138.1) | -34.9 (-59.4, -0.6) |
|  |  | DHA | 43.4 (-11.8, 118.7) | -31 (-56.7, 4.2) |
|  |  | EPA | 90.4 (6.8, 210.9) | -34.7 (-62.1, 5.1) |
| Pathway | |  |  |  |
| Fatty acid | Enzyme | Eicosanoid |  |  |
| LA | CYP | 9,10-EpOME | 8.7 (-18, 42.1) | -4.2 (-26.2, 23.1) |
|  |  | 9,10-DiHOME | 6.1 (-32.2, 57.3) | 7.1 (-28.9, 55.9) |
|  |  | 12,13-EpOME | -3 (-28.5, 30) | 39.3 (4.3, 83.4) |
|  |  | 12,13-DiHOME | 31.6 (-28.5, 123.7) | 19.4 (-32.3, 95.6) |
|  | LOX | 13-HODE | 18.6 (-7.3, 49.2) | -0.4 (-21.1, 25.1) |
|  |  | 9-HODE | 16.2 (-11.4, 49.6) | 5.2 (-19.1, 34.5) |
| AA | CYP | 5,6-DHET | 48.8 (7.4, 100.5) | 4.2 (-22.4, 38.5) |
|  |  | 8,9-DHET | 42.5 (9.3, 80.2) | -3.6 (-24.2, 21.3) |
|  |  | 11,12-DHET | 21.7 (3.5, 42.5) | 6.9 (-7.8, 23.7) |
|  |  | 14,15-DHET | 29.5 (10, 50.8) | -7.2 (-20.4, 7.1) |
|  |  | 19-HETE | 20.5 (-6.8, 53.6) | 2 (-19.3, 27.4) |
|  |  | 20-HETE | 35.6 (1.3, 77.6) | 4.6 (-20.7, 34.2) |
|  | LOX | 5-HETE | 49.6 (-8.3, 128.9) | -21.8 (-51.2, 18.9) |
|  |  | 8-HETE | 46 (-10.3, 124.6) | -15.4 (-46.6, 30.4) |
|  |  | 11-HETE | 36.1 (-7, 92.9) | -15.2 (-41.1, 19.7) |
|  |  | 12-HETE | 61.5 (5.3, 135.1) | -14.1 (-41.7, 24.5) |
|  |  | 15-HETE | 42.1 (2.2, 90.8) | -13 (-36.2, 15.8) |
|  | COX | PGE_2_ | 32.4 (-36.7, 147.1) | -3.3 (-51.8, 75.2) |
|  |  | TXB_2_ | -19.1 (-53, 30.5) | 9.6 (-34.8, 74.5) |
| DHA | CYP | 7,8-DiHDPA | 23.5 (-19.9, 83.2) | 2.7 (-32.9, 51.5) |
|  |  | 10,11-DiHDPA | 29.5 (-4.4, 70.3) | -2.5 (-26.7, 27.4) |
|  |  | 13,14-DiHDPA | 13.7 (-9.8, 42.1) | 1.5 (-18.5, 25.9) |
|  |  | 16,17-DiHDPA | 11.7 (-12.4, 39.9) | -6.5 (-25.2, 15.1) |
|  |  | 19,20-DiHDPA | 11.1 (-9.4, 33.8) | -4.9 (-21.4, 13.9) |
|  |  | 19,20-EpDPE | 14.7 (-18.9, 56.8) | -6.3 (-32.7, 27.7) |
| EPA | CYP | 14,15-DiHETE | 33 (-2.7, 77.4) | -19.8 (-40.1, 5.2) |
|  |  | 17,18-DiHETE | 22.1 (-4.6, 53.4) | -15 (-31.9, 5.1) |

^a^ Bayesian linear mixed models were used to estimate the standardized population mean concentrations of maternal plasma fatty acids and eicosanoids in small for gestational age (SGA) and large for gestational age (LGA) cases compared to adequate growth controls (referent).

^b^ Bayesian models were adjusted for maternal age (continuous), race (White, Black, other), pre-pregnancy BMI (continuous), and insurance status (private, public). Models included participant-specific random intercepts and penalized splines on gestational age. Biomarker concentrations were log_2_-transformed prior to modeling and standardized population means of BLMs were estimated to calculate percent differences between fetal growth outcome categories.

^c^ A total of 13 participants had a relevant comorbidity, which included preterm birth, chronic hypertension, gestational hypertension, preeclampsia, or gestational diabetes mellitus. One participant was missing plasma samples.
