# Supplementary material for: Longitudinal profiles of plasma eicosanoids during pregnancy and size for gestational age at delivery: A nested case-control study
Source: PLoS Med. 2020 Aug 14;17(8):e1003271. doi: 10.1371/journal.pmed.1003271 (PMC7428021; doi:10.1371/journal.pmed.1003271)
Supplement: S7 Table — (DOCX) [file pmed.1003271.s013.docx]

**S7 Table. Characteristics of participants with non-missing and missing eicosanoid values: n(%) or median (25^th^, 75^th^ percentiles).**

|  | Overall study population  (N=90) | No missing  measurements  (n=80) | Missing at least 1 measurement  (n=10) |
| --- | --- | --- | --- |
| Matched variables |  |  |  |
| Maternal age (years) | 33.2 (29.5, 37.3) | 33.1 (29.8, 37.3) | 34.9 (29.2, 36.2) |
| Maternal race |  |  |  |
| White | 54 (60) | 50 (63) | 4 (40) |
| Black | 18 (20) | 15 (19) | 3 (30) |
| Other | 18 (20) | 15 (19) | 3 (30) |
| Pre-pregnancy BMI (kg/m^2^) | 23.5 (21.4, 27.5) | 23.6 (21.4, 27.7) | 22.7 (21.7, 26.5) |
| Gestational age at delivery (weeks) | 38.7 (37.9, 39.4) | 38.9 (38.0, 39.5) | 38.1 (37.4, 38.6) |
| Non-matched variables |  |  |  |
| Gestational age at sample collection (weeks) |  |  |  |
| Visit 1 | 11.1 (9, 12.9) | 11.1 (8.9, 12.7) | 11.8 (11.1, 14.6) |
| Visit 2 | 25.6 (24.9, 26.3) | 25.6 (24.9, 26.3) | 26.9 (25.1, 27.8) |
| Visit 3 | 35 (34.4, 35.7) | 35 (34.4, 35.7) | 36.9 (34.1, 37) |
| Birth weight (kg) | 3.2 (2.4, 4.0) | 3.2 (2.4, 4.1) | 2.6 (2.2, 3.6) |
| Birth weight percentile (%) | 33.0 (3.8, 93.6) | 35.0 (3.8, 94.7) | 12.0 (3.3, 87.3) |
| Health insurance |  |  |  |
| Private | 66 (73) | 59 (74) | 7 (70) |
| Public | 24 (27) | 21 (26) | 3 (30) |
| Maternal education |  |  |  |
| High school or less | 7 (8) | 6 (8) | 1 (10) |
| Some college/  technical school | 22 (24) | 21 (26) | 1 (10) |
| College graduate or  more | 61 (68) | 53 (66) | 8 (80) |
| Parity |  |  |  |
| Nulliparous | 24 (27) | 23 (29) | 1 (10) |
| Parous | 66 (73) | 57 (71) | 9 (90) |
| Smoking during  pregnancy (yes) | 6 (7) | 6 (8) | 0 (0) |
